# Ketoreductase TpdE from *Rhodococcus jostii* TMP1: characterization and application in the synthesis of chiral alcohols

**DOI:** 10.7717/peerj.1387

**Published:** 2015-11-10

**Authors:** Jonita Stankevičiūtė, Simonas Kutanovas, Rasa Rutkienė, Daiva Tauraitė, Romualdas Striela, Rolandas Meškys

**Affiliations:** 1 Department of Molecular Microbiology and Biotechnology, Institute of Biochemistry, Vilnius University, Vilnius, Lithuania; 2 Department of Organic Chemistry, Institute of Chemistry of Center for Physical Sciences and Technology, Vilnius, Lithuania

**Keywords:** Ketoreductase TpdE, Diacetyl, Butan-3-one-2-yl alkanoates, Bioconversion, Asymmetric reduction

## Abstract

**Background.** Production of highly pure enantiomers of vicinal diols is desirable, but difficult to achieve. Enantiomerically pure diols and acyloins are valuable bulk chemicals, promising synthones and potential building blocks for chiral polymers. Enzymatic reduction of ketones is a useful technique for the synthesis of the desired enantiomeric alcohols. Here, we report on the characterization of a ketoreductase TpdE from *Rhodococcus jostii* TMP1 that is a prospective tool for the synthesis of such compounds.

**Results.** In this study, NADPH-dependent short-chain dehydrogenase/reductase TpdE from *Rhodococcus jostii* TMP1 was characterized. The enzyme exhibited broad substrate specificity towards aliphatic 2,3-diketones, butan-3-one-2-yl alkanoates, as well as acetoin and its acylated derivatives. TpdE stereospecifically reduced *α*-diketones to the corresponding diols. The GC-MS analysis of the reduction products of 2,3- and 3,4-diketones indicated that TpdE is capable of reducing both keto groups in its substrate leading to the formation of two new chiral atoms in the product molecule. Bioconversions of diketones to corresponding diols occurred using either purified enzyme or a whole-cell *Escherichia coli* BL21 (DE3) biocatalyst harbouring recombinant TpdE. The optimum temperature and pH were determined to be 30–35 °C and 7.5, respectively.

**Conclusions.** The broad substrate specificity and stereoselectivity of TpdE from *Rhodococcus jostii* TMP1 make it a promising biocatalyst for the production of enantiomerically pure diols that are difficult to obtain by chemical routes.

## Introduction

Compared to other chiral compounds, diols receive particular attention because, due to the existence of two asymmetric centers, they can be further transformed into a variety of complex chiral systems (Chen, Chen & Wu, 2012). Enantiomerically pure diols and acyloins are valuable bulk chemicals, as well as promising synthones, due to their extensive applications in agrochemical, fine chemical and food industries or in the synthesis of pharmaceuticals (Zhu, Yang & Hua, 2006; Xiao et al., 2010; Calam et al., 2013). Furthermore, optically active diols are potential building blocks for chiral polymers (Bennet & San, 2001). Since chemical synthesis or fermentations generally lead to the production of racemic mixtures, enzymatic reduction of ketones is a useful technique for the stereoselective synthesis of non-racemic alcohols (Zhu, Yang & Hua, 2006). A broad range of alcohol dehydrogenases used for the asymmetric reduction of prochiral ketones have been already described (Zhu, Yang & Hua, 2006; Yan, Lee & Liao, 2009; Yu et al., 2011; Chen, Chen & Wu, 2012; Schweiger et al., 2013; Chen et al., 2014). Recently, great attention has been paid to the studies on stereospecific acetoin(diacetyl) reductases (ADRs, also known as 2,3-butanediol dehydrogenases), which are key enzymes in the microbial production of 2,3-butanediol (Celinska & Grajek, 2009; Calam et al., 2013; Gao et al., 2013; Wang et al., 2014; Zhang et al., 2014). The latter compound has two stereogenic centers and three types of stereoisomers: *meso*, 2*R*, 3*R*, and 2*S*, 3*S*. Stereospecific ADRs can catalyze the reduction of diacetyl to acetoin and then to optically pure 2,3-butanediol (Wang et al., 2014). To date, several *Rhodococcus* strains have been reported to possess oxidoreductases that catalyze the asymmetric reduction of prochiral aliphatic and aromatic ketones to chiral alcohols (Edegger et al., 2006; Yang et al., 2012; Wang et al., 2014). Nevertheless, there is still a great demand for biocatalysts suitable for the efficient stereoselective production of diols and acyloins.

**Figure 1:**
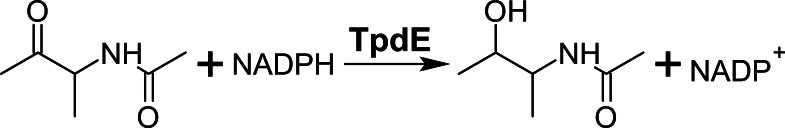
Reduction of *N*-(butan-3-one-2-yl)acetamide into *N*-(3-hydroxybutan-2-yl)acetamide by TpdE.

Previously, we have shown that the protein TpdE from *Rhodococcus jostii* TMP1 is involved in the catabolism of tetramethylpyrazine, where it reduces *N*-(butan-3-one-2-yl)acetamide (Fig. 1) (Kutanovas et al., 2013). Here we provide a biochemical characterization of TpdE. We also demonstrate that both purified TpdE and whole-cells producing recombinant TpdE are capable of stereoselective reduction of various 2,3- and 3,4-diketones, acetoin and its acylated derivatives.

## Materials and Methods

### Reagents

All chemicals used in the study were of analytical grade. The reagents were purchased from Sigma-Aldrich (Germany), unless stated otherwise. Ester group bearing acetoin derivatives (butan-3-one-2-yl alkanoates) were synthesized using *N*, *N*-dicyclohexylcarbodiimide and 4-dimethylaminopyridine as catalyst according to reference (Neises & Steglich, 1978). The compounds were purified by column chromatography (silica gel, eluent hexane/chloroform). The structure and the purity of the synthesized compounds were varified by NMR spectroscopy and HPLC-MS.

*Butan-3-one-2-yl picolinate*^1^H NMR (400 MHz, CDCl_3_, ppm): *δ* = 1.63 (d, *J* = 7.1 Hz, 3H, CH_3_), 2.28 (s, 3H, CH_3_), 5.42 (q, *J* = 7.1 Hz, 1H, CH), 7.55 (ddd, *J* = 7.8, 4.8, 1.1 Hz, 1H, CH), 7.91 (td, *J* = 7.8, 1.8 Hz, 1H, CH), 8.19 (dt, *J* = 7.8, 1.1 Hz, 1H, CH), 8.83 (ddd, *J* = 4.8, 1.8, 1.1 Hz, 1H, CH). ^13^C NMR (100 MHz, CDCl_3_, ppm): *δ* = 205.03, 164.42, 149.45, 147.20, 137.31, 127.31, 125.56, 74.72, 25.76, 16.11. MS (ESI^+^), *m*/*z* 194 [M+H]^+^. Yield 91%.

*Butan-3-one-2-yl nicotinate*^1^H NMR (400 MHz, CDCl_3_, ppm): *δ* = 1.61 (d, *J* = 7.1 Hz, 3H, CH_3_), 2.28 (s, 3H, CH_3_), 5.39 (q, *J* = 7.1 Hz, 1H, CH), 7.58 (ddd, *J* = 8.0, 5.0, 0.9 Hz, 1H, CH), 8.49 (dt, *J* = 8.0, 2.2, 1.7 Hz, 1H, CH), 8.87 (dd, *J* = 5.0, 1.7 Hz, 1H, CH), 9.32 (dd, *J* = 2.2, 0.9 Hz, 1H, CH). ^13^C NMR (100 MHz, CDCl_3_, ppm): *δ* = 204.33, 163.83, 151.92, 149.39, 138.95, 126.22, 124.12, 74.68, 25.79, 16.05. MS (ESI^+^), *m*/*z* 194 [M+H]^+^. Yield 87%.

*Butan-3-one-2-yl isonicotinate*^1^H NMR (400 MHz, CDCl_3_, ppm): *δ* = 1.61 (d, *J* = 7.1 Hz, 3H, CH_3_), 2.28 (s, 3H, CH_3_), 5.40 (q, *J* = 7.1 Hz, 1H, CH), 8.03–8.06 (m, 2H, CH), 8.86 – 8.88 (m, 2H, CH). ^13^C NMR (100 MHz, CDCl_3_, ppm): *δ* = 203.92, 163.74, 148.86, 138.39, 123.74, 74.71, 25.81, 15.99. MS (ESI^+^), *m*/*z* 194 [M+H]^+^. Yield 89%.

3-Hydroxybutan-2-yl nicotinate enantiomers were synthesized from nicotinic acid and enantiomerically pure 2,3-butanediol. To a solution of 1.22 mmol of nicotinic acid in 5 ml dichloromethane, 2.44 mmol of *N*, *N*′-dicyclohexylcarbodiimide, 0.61 mmol of 4-dimethylaminopyridine and 1.83 mmol of appropriate enantiomerically pure 2*R*, 3*R*-, 2*S*, 3*S*- or *meso*-butanediol were added. The reaction mixture was stirred at room temperature overnight. The resulting precipitate was filtered and washed several times with dichloromethane. The solvent was removed under reduced pressure, and the crude mixture was purified by column chromatography (silica gel, chloroform/methanol mixture, 10:0 → 10:0.5). The solvents were removed under reduced pressure to afford colourless oily reaction products.

*(2R,3R)-3-hydroxybutan-2-yl nicotinate*^1^H NMR (400 MHz, DMSO-d_6_, ppm): *δ* = 1.11 (d, *J* = 6.4 Hz, 3H, CH_3_), 1.25 (d, *J* = 6.4 Hz, 3H, CH_3_), 3.76 (m, 1H, CH), 4.93 (m, 2H, CH, OH), 7.57 (ddd, *J* = 8.0, 4.8, 0.9 Hz, 1H, CH), 8.33 (ddd, *J* = 8.0, 2.2, 1.8 Hz, 1H, CH), 8.81 (dt, *J* = 4.8, 2.4 Hz, 1H, CH), 9.14 (dd, *J* = 2.2, 0.9 Hz, 1H, CH). ^13^C NMR (100 MHz, DMSO-d_6_, ppm): *δ* = 164.82, 154.00, 150.61, 137.37, 126.50, 124.27, 75.65, 68.02, 18.64, 15.95. MS (ESI^+^), *m*/*z* 196 [M+H]^+^. Yield 59%.

*(2S,3S)-3-hydroxybutan-2-yl nicotinate*^1^H NMR (400 MHz, DMSO-d_6_, ppm): *δ* = 1.11 (d, *J* = 6.4 Hz, 3H, CH_3_), 1.25 (d, *J* = 6.4 Hz, 3H, CH_3_), 3.76 (m, 1H, CH), 4.93 (m, 2H, CH, OH), 7.57 (m, 1H, CH), 8.33 (ddd, *J* = 8.0, 2.2, 1.8 Hz, 1H, CH), 8.81 (dt, *J* = 4.8, 2.4 Hz, 1H, CH), 9.14 (dd, *J* = 2.2, 0.9 Hz, 1H, CH). ^13^C NMR (100 MHz, DMSO-d_6_, ppm): *δ* = 164.81, 153.99, 150.61, 137.36, 126.50, 124.26, 75.65, 68.02, 18.65, 15.94. MS (ESI^+^), *m*/*z* 196 [M+H]^+^. Yield 65%.

*Mixture of 2R,3S- and 2S,3R-3-hydroxybutan-2-yl nicotinate*^1^H NMR (400 MHz, DMSO-d_6_, ppm): *δ* = 1.13 (d, *J* = 6.4 Hz, 3H, CH_3_), 1.25 (d, *J* = 6.4 Hz, 3H, CH_3_), 3.80 (m, 1H, CH), 4.93 (m, 2H, CH, OH), 7.57 (ddd, *J* = 8.0, 4.8, 0.9 Hz, 1H, CH), 8.32 (ddd, *J* = 8.0, 2.2, 1.8 Hz, 1H, CH), 8.82 (dd, *J* = 4.8, 1.8 Hz, 1H, CH), 9.13 (m, 1H, CH). ^13^C NMR (100 MHz, DMSO-d_6_, ppm): *δ* = 164.73, 154.01, 150.57, 137.34, 126.54, 124.28, 75.92, 68.14, 18.99, 15.35. MS (ESI^+^), *m*/*z* 196 [M+H]^+^. Yield 76%.

3-Hydroxybutan-2-yl nicotinate enantiomers were used as HPLC standards for the analysis of enantiomeric ratio in enzymatic and chemical reduction reactions.

### Chemical reduction of butan-3-one-2-yl nicotinate

To a solution of 0.2 mmol of butan-3-one-2-yl nicotinate in a mixture of 0.5 ml water and 2.5 ml ethanol 0.1 mmol of NaBH_4_ was added. The reaction mixture was stirred at room temperature for 15 min. The solvent was removed under reduced pressure, and the crude mixture was purified by column chromatography (silica gel, chloroform/methanol mixture, 10:0 → 10:0.5). The solvents were removed under reduced pressure to afford colourless oil reaction product.

*3-Hydroxybutan-2-yl nicotinate (racemate)*^1^H NMR (400 MHz, DMSO-d_6_, ppm): *δ* = 1.11 and 1.13 (2d, *J* = 6.4 Hz, 3H, CH_3_), 1.25 and 1.26 (2d, *J* = 6.4 Hz, 3H, CH_3_), 3.78 (m, 1H, CH), 4.93 (m, 2H, CH, OH), 7.58 (ddd, *J* = 8.0, 4.8, 0.9 Hz, 1H, CH), 8.33 (m, 1H, CH), 8.82 (ddd, *J* = 4.8, 1.7, 0.9 Hz, 1H, CH), 9.14 (ddd, *J* = 4.2, 2.2, 0.9 Hz, 1H, CH). ^13^C NMR (100 MHz, DMSO-d_6_, ppm): *δ* = 164.90, 154.23, 150.78, 137.50, 126.55, 124.30, 75.95, 68.21, 18.86, 15.33. MS (ESI^+^), *m*/*z* 196 [M+H]^+^. Yield 52%.

### Sequence analysis

Phylogenetic analysis was performed by aligning the amino acid sequence of TpdE with known characterized acetoin reductases/2,3-butanediol dehydrogenases or with the amino acid sequences of its closest relatives in non-redundant protein sequences database using BLASTP (http://www.ncbi.nlm.nih.gov/blast/). A phylogenetic tree was constructed and displayed using the neighbor-joining method with MEGA6 (Tamura et al., 2013).

### Expression and purification of the recombinant TpdE

To obtain a large amount of TpdE, the overexpression of the recombinant protein was performed as described previously (Kutanovas et al., 2013) with slight modifications. *E. coli* BL21 (DE3) cells harbouring pET-*tpdE* were grown in BHI medium (supplemented with 50 mg/ml ampicillin) to an OD_600_ of 0.8. The protein expression was induced with 0.1 mM IPTG, and the culture was incubated at 30 °C for 17 h. Cells were harvested by centrifugation, and the pellet was resuspended in 50 mM potassium phosphate buffer (pH 7.2) containing 0.1 M NaCl. The cell suspension was disrupted by sonication, and the crude cell extract was centrifuged for 10 min at 3220 rcf. The TpdE tagged with C-(His)_6_ was purified from clear supernatant by affinity chromatography using Ni^2+^-chelating column (GE Healthcare Bio-Sciences, Sweden) (Fig. S1) and desalted by dialysis in 50 mM potassium phosphate buffer (pH 7.2) or using a HiTrap Desalting column (GE Healthcare Bio-Sciences, Sweden). Proteins were separated on 14% SDS-PAGE gel and visualized by Coomassie blue staining.

### Activity measurements of TpdE

Keto group reductions or alcohol oxidations were determined by following either the oxidation of NADPH or the reduction of NADP^+^ at 340 nm, using a Helios gamma UV-visible spectrophotometer (Thermo Fisher Scientific). Each reduction reaction mixture consisted of 0.2 mM NADPH, 10 mM substrate and 50 mM phosphate buffer (pH 7.2). Unless stated otherwise, all reactions were performed at 30 °C using diacetyl as substrate. The initial reaction rate was recorded. One unit of TpdE activity was defined as an amount of the enzyme that catalyzed the oxidation of 1 µmol of NADPH per minute under the conditions of the assay. Each oxidation reaction consisted of 0.2 mM NADP^+^, 10 mM (2*S*, 3*S*)-(+)-2,3-butanediol, (2*R*, 3*R*)-(−)-2,3-butanediol or *meso*-2,3-butanediol as substrate and 50 mM sodium bicarbonate buffer solution (pH 9.1). The reactions were started by the addition of the substrate.

The effect of metal ions on TpdE activity was investigated using different metal salts (CaCl_2_, CoCl_2_, CuCl_2_, MgCl_2_, MnCl_2_, NiCl_2_, and ZnCl_2_) at a final concentration of 1 mM. The residual activity of the TpdE was measured as described above. The activity of TpdE without addition of metal ions was defined as 100%.

### Determination of optimal pH for TpdE

The pH optimum of the enzyme was determined using the following buffer systems: sodium citrate (pH 4.5–6.0), Tris-HCl (pH 5.7–8.5), sodium phosphate (pH 6–8), sodium succinate (pH 4–6), glycine-NaOH (pH 8.6–9.5) and MES (pH 5.3–6.8). The concentration of the buffers was 50 mM. The activity was assayed spectrophotometrically by measuring a decrease in absorption at 340 nm resulting from oxidation of NADPH at 30 °C. In all reactions diacetyl was used as the second substrate.

### Determination of optimal temperature for TpdE

To determine the effect of temperature on TpdE activity, reactions were carried out in the standard reaction mixture at different temperatures ranging from 15 to 50 °C, at pH 7.2. The thermostability of the TpdE was investigated at 25–50 °C. The enzyme solution was kept at different temperatures in 50 mM phosphate buffer (pH 7.2) for 10 min and then immediately cooled on ice. The residual activity was measured at 30 °C as described previously.

### Determination of kinetic parameters

Kinetic measurements of substrate reduction were performed spectrophotometrically using a computer-controlled Helios gamma UV–visible spectrophotometer (Thermo Fisher Scientific) in 50 mM phosphate buffer (pH 7.2). The kinetic curves were recorded by the decrease in absorption of NADPH (ε_340_ = 6,220 M^−1^ cm^−1^) at 30 °C in the presence of TpdE and an appropriate carbonyl. The reaction was started by adding the solution of the enzyme. Kinetic parameters for the diketones and acetoin derivatives were determined by varying their concentrations up to 100 mM, the concentration of NADPH was constant (0.2 mM). The initial rates (*v*_0_) of the reduction of different substrates were calculated by fitting the kinetic curves with the linear function. For the linear dependence, the initial rate was calculated as a slope. To analyze the dependence of *v*_0_ on the substrate concentration and determine the apparent kinetic parameters *V*_max_ and *K_M_*, the Michaelis–Menten equation was used. For data fitting, the program GraFit (Erithacus Software LTD) was used. Catalytic constants (*k*_cat_) were calculated as a ratio of *V*_max_ and the total concentration of TpdE. Specificity constants of the enzyme and substrates were calculated as a ratio of *k*_cat_ and *K_M_*.

### GC-MS analysis

GC-MS analysis was performed with a Shimadzu GCMS-QP2010Ultra Plus (Kyoto, Japan). Chromatographic separation was achieved on a Rtx^®^-1701 column (30 m × 0.25 mm I.D., 0.25 µm film thickness with 5 m Integra-guard; Restek, USA) using helium as carrier gas at 40 cm/s in a constant linear velocity mode. The GC oven temperature was initially increased from 80 °C to 250 °C at a rate of 15 °C/min and holding at 250 °C for 2.7 min, giving a total run time of 15 min. The temperatures of injector, interface, and ion source were 250, 270, and 220 °C, respectively. Detection was operated by selected ion monitoring (SIM) mode (70 eV, electron impact mode).

In the SIM mode, the peaks were identified by matching the retention time and the abundant ions (*m*/*z*). The injection volume was 0.3 µL. Data was collected and analyzed using the GC-MS solution version 2.71 (Kyoto, Japan). Before a GC-MS analysis the samples were dried and dissolved in ethyl acetate.

### GC analysis

GC analysis of 2,3-butanediol stereoisomers was performed with a Shimadzu GC-2010 Plus with FID detector (Kyoto, Japan). Chromatographic separation was achieved on a Rt-bDEXsm column (30 m × 0.25 mm I.D., 0.25 µm film thickness; Restek, USA) using hydrogen as carrier gas (flow rate 2 ml/min) in a constant linear velocity mode. The GC oven temperature was initially set at 40 °C and kept for 5 min, then increased to 160 °C at a rate of 15 °C/min and kept for the final 5 min. The temperature was set at 230 °C for the injector and the detector. The injection volume was 0.2 µL. Data was collected and analyzed using the GCsolution version 2 (Kyoto, Japan). Before a GC analysis the samples were dried and dissolved in methanol.

Enantio purity of (2*S*, 3*S*)-2,3-butanediol was defined as 100%[S]/([S]+[M]+[R]). Diastereomeric excess *de* = 100%([S]−[M])/([S]+[M]). [S], [M] and [R] represent the concentrations of (2*S*, 3*S*)-2,3-butanediol, *meso*-2,3-butanediol and (2*R*, 3*R*)-2,3-butanediol, respectively.

### HPLC analysis

HPLC analysis was performed using a high performance liquid chromatography system (CBM-20A controller, two LC-2020AD pumps, SIL-30AC auto sampler and CTO-20AC column oven; Shimadzu, Japan) equipped with a photo diode array (PDA) detector (SPD-M20A Prominence diode array detector; Shimadzu, Japan). The chromatographic separation was conducted using a Chiralcel OD-H column, 4.6 × 250 mm (Daicel, France), at 30 °C and a mobile phase *n*-hexane/isopropanol (16:1) delivered in the isocratic elution mode (0.5 ml/min). The data was analyzed using the LabSolutions LCMS software. Before an HPLC analysis the samples were dried and dissolved in the mixture of *n*-hexane/isopropanol (16:1).

### Whole-cell bioconversion

Biomass of *E. coli* BL21 (DE3) pTpdE cells grown overnight in 200 ml of NB medium was collected by centrifugation, washed twice with 0.9% NaCl and suspended in 100 ml of 10 mM potassium phosphate buffer, pH 7.2. The suspension was supplemented with 0.25% (w/v) glucose and with 0.05% (w/v) of corresponding substrate. The suspension was incubated at 30 °C for 20 h. *E. coli* BL21 (DE3) cells carrying empty pET vector were used as a control. The process of the conversion of keto esters was followed by HPLC. The products of diketone biotransformation were analyzed by GC or GC-MS.

### Determination of diacetyl concentration

Diacetyl was derivatized with creatine in the presence of *α*-naphthol. Diacetyl containing samples were mixed with 1.6 mM creatine, 30 mM *α*-naphthol and 300 mM KOH. Water solutions of creatine and KOH were used for derivatization, while *α*-naphthol was freshly prepared in dimethyl sulfoxide. Reaction mixture was incubated at 25 °C for 30 min. The absorbance was registered at 540 nm using the reagent blank to null the spectrophotometer. The calibration curve was developed using a known serial concentration samples of derivatized diacetyl. The concentration of diacetyl in bioconversion mixtures was assigned by comparison with calibration curve.

## Results and Discussion

### Bioinformatic analysis of TpdE

**Figure 2:**
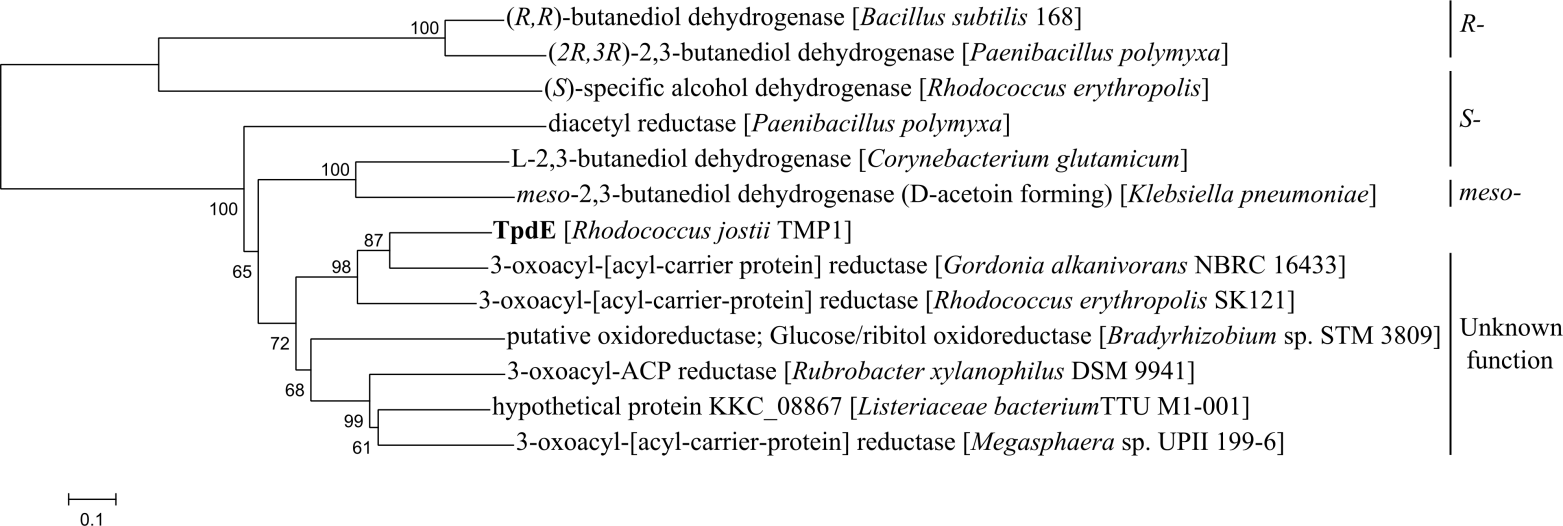
Phylogenetic analysis of amino acid sequence of TpdE from *Rhodococcus jostii* TMP1. The tree was constructed by the program MEGA 6 using the neighbor-joining method.

Comparative amino acid sequence analysis revealed that TpdE from *Rhodococcus jostii* TMP1 has the highest similarity to 3-oxoacyl-[acyl-carrier proteins] reductases (Fig. 2). The TpdE contained conserved amino acid residues characteristic of all “classical” short-chain dehydrogenases/reductases (SDRs), wherein the glycine-rich coenzyme binding motif T_15_G_16_XXXG_20_XG_22_ was conserved in the *N*-terminal region (Oppermann et al., 2003). The conserved catalytic tetrad Asn119-Ser148-Tyr161-Lys165 was also identified. Therefore, it can be stated that TpdE is a 3-oxoacyl-[acyl-carrier protein] reductase, which belongs to the SDR family. Moreover, the TpdE has a NAD(P)H/NAD(P)^+^-binding Rossmann fold domain that is also common among members of SDR superfamily. Notwithstanding that TpdE has low sequence identity (<37%) to SDRs with known functions, the length of the amino acid chain as well as the presence of conserved cofactor-binding and active site motifs suggests that this protein may be assigned to the family of “classical” SDRs.

### TpdE substrate range and biochemical characterization

**Figure 3:**
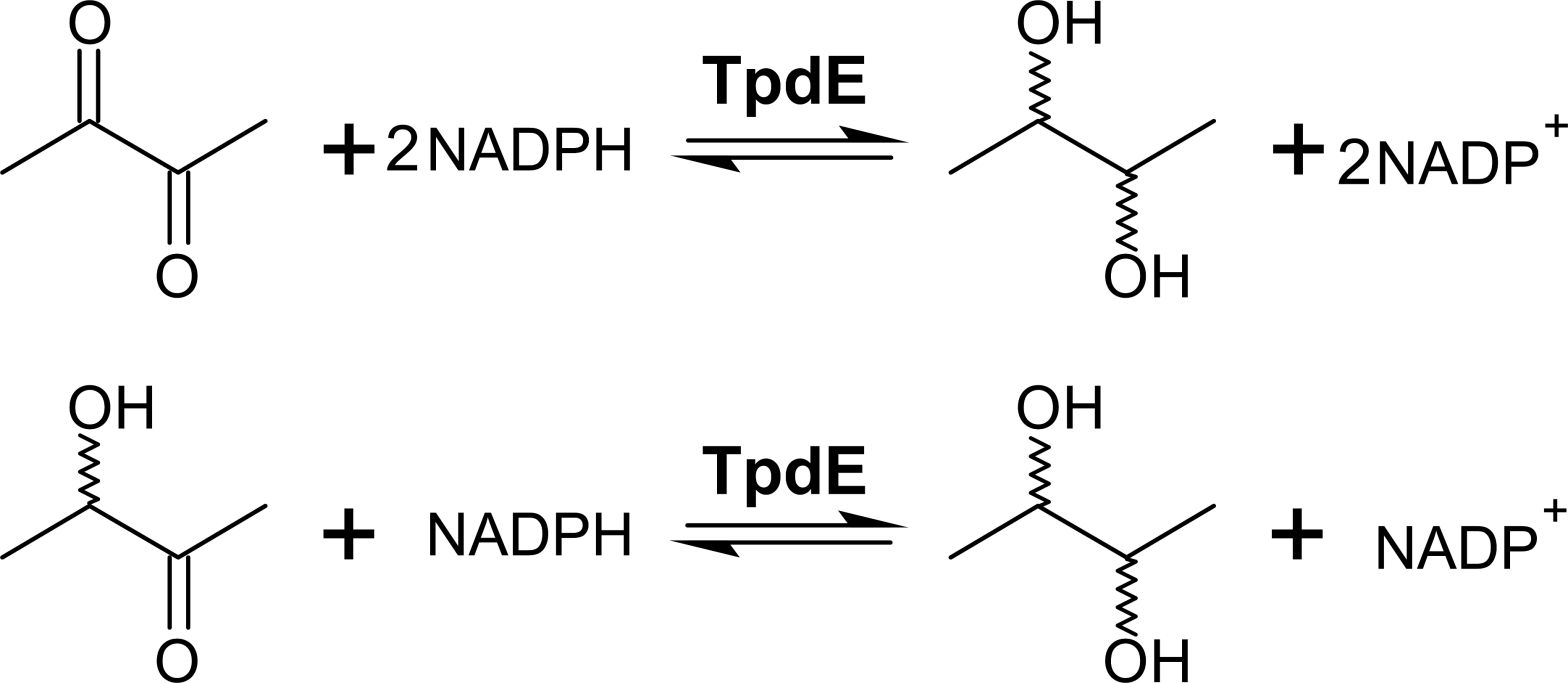
Diacetyl conversion to 2,3-butanediol catalyzed by TpdE.

We previously showed that TpdE is able to reduce carbonyl group of *N*-(butan-3-one-2-yl)acetamide (Kutanovas et al., 2013). In this study we demonstrated that TpdE is also capable of reducing diacetyl when NADPH was used as a co-substrate. To elucidate if one or both keto groups of diacetyl are reduced by TpdE, the reaction was investigated spectrophotometrically using diacetyl or acetoin as a substrate. As shown in Fig. 3, TpdE catalyzed reduction of both substrates, thus the formation of 2,3-butanediol occurred.

The effect of pH on the activity of TpdE was investigated within a range of 4.0–9.5 at 30 °C (Fig. S2). The results revealed that the optimum pH for the reduction of diacetyl was about 7.5, and TpdE retained more than 60% of its maximum activity over the pH range 6.5–8.0. In addition, TpdE was most active when assayed in 50 mM sodium phosphate or 50 mM Tris-HCl buffer solutions. Moreover, we noticed that the activity of TpdE increased in the presence of EDTA suggesting that divalent metal ions may affect the enzyme. For this reason, the influence of metal ions on the activity of TpdE was studied (Fig. S3). The results obtained indicated that the addition of CaCl_2_, MgCl_2_ or MnCl_2_ had no effect on TpdE, while the presence of Co^2+^, Cu^2+^, Ni^2+^ or Zn^2+^ ions reduced the enzymatic activity to 26%, 4%, 54% and 51%, respectively.

The effect of temperature on the activity and stability of TpdE was then examined (Fig. S4). Maximum activity of TpdE was observed at 35–40 °C, while further increase in temperature resulted in a sharp decrease in enzyme activity that could be associated with the disruption of the native protein structure. Thermostability of TpdE was investigated in the range of 25–50 °C. After incubation at 40 °C for 10 min, about 70% of the enzyme activity was lost. Moreover, the incubation at 45 °C and higher temperatures caused an almost complete inactivation of the enzyme. These results suggested that the irreversible denaturation of TpdE occurs at temperatures higher than 35 °C. It can be summarized that the optimal temperature for the activity of TpdE is in the range 30–35 °C. At 40 °C the reaction proceeds faster than at 30 °C yet the enzyme becomes unstable. Thus, the increased reaction rate does not compensate for the activity loss resulting from protein denaturation.

### TpdE—a promising tool for the production of chiral alcohols

**Figure 4:**
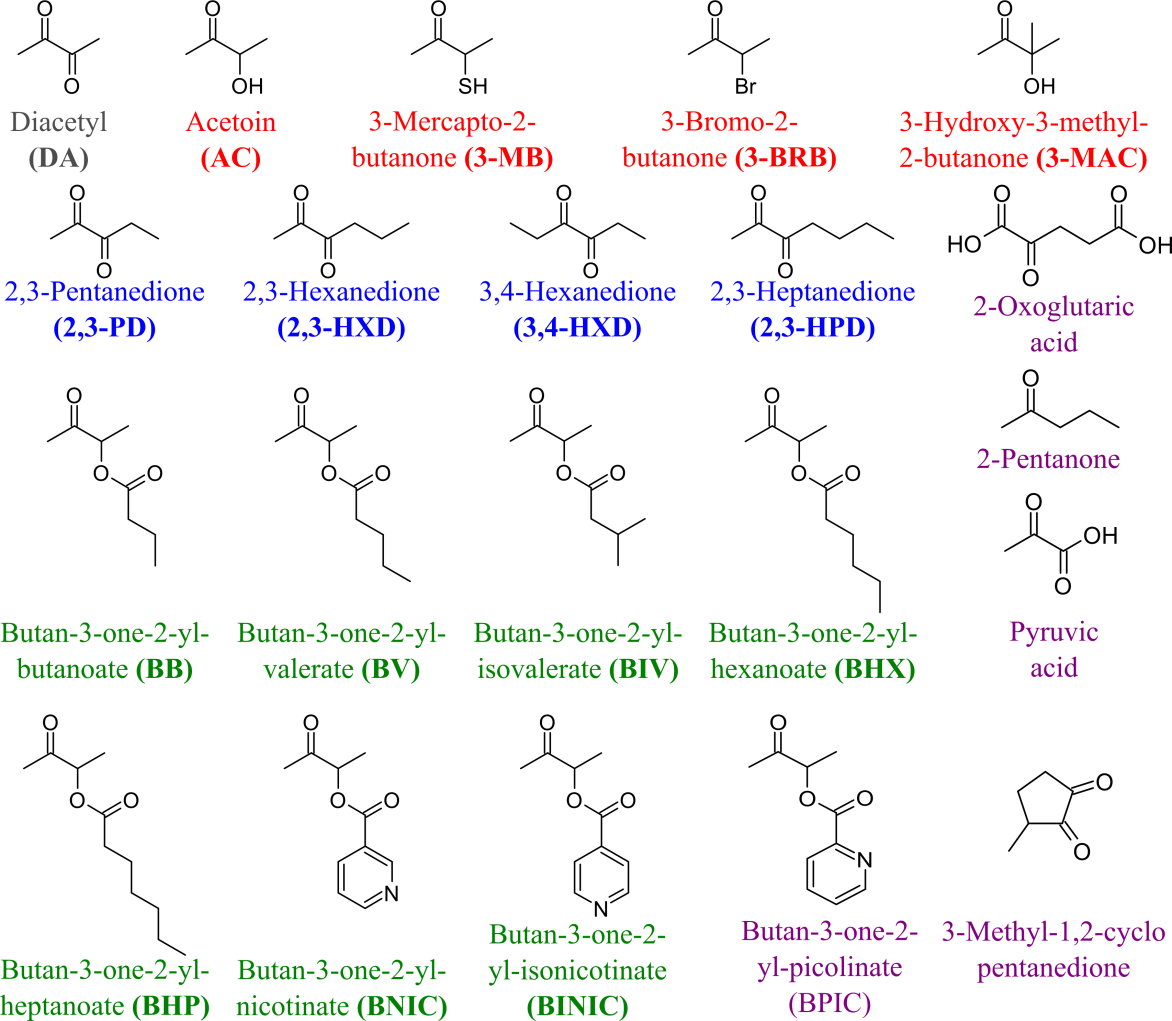
The compounds used as potential TpdE substrates. Red are acetoin and its acylated derivatives; blue, diketones; green, keto esters; grey, diacetyl; purple are compounds that have not been reduced by TpdE.

**Figure 5:**
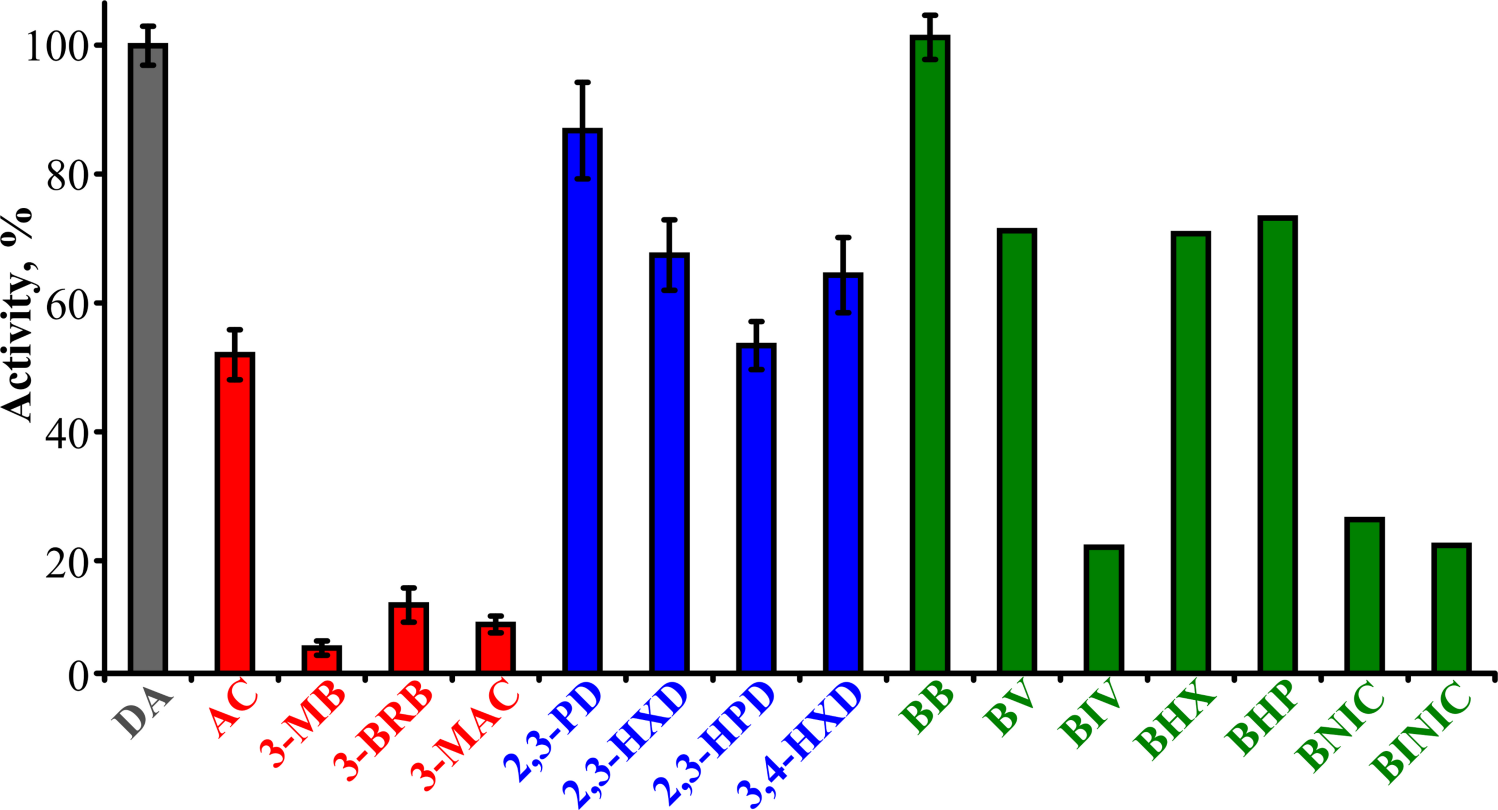
The dependence of TpdE activity on the substrate used. TpdE activity was measured in phosphate buffer (50 mM, pH 7.2) containing 0.2 mM NADPH and 10 mM of corresponding substrate. 100% is equal to the activity of TpdE using diacetyl as substrate.

To determine the substrate specificity of TpdE, various ketones (having a carbon chain length ranging from 4 to 7), acetoin derivatives and keto esters were tested as potential substrates (Fig. 4). As seen in Fig. 5, TpdE displayed the highest activity with diacetyl among diketones, and reduced 2,3- and 3,4-diketones in a chain length-dependent manner: the longer the aliphatic chain, the slower the reaction. In addition, the enzyme accepted 3-mercapto-2-butanone, 3-bromo-2-butanone, and 3-hydroxy-3-methyl-2-butanone as substrates though with a relatively low conversion rate. Previously, enantiomers of 3-bromo-2-butanols were prepared by application of lipases (Liu, Berglund & Hogberg, 2005). However, enzymatic conversion of acetoin derivatives received limited attention yet. Our results demonstrated that TpdE has a broad substrate specificity (Fig. 5) and is applicable for the synthesis of various 2-butanols from corresponding ketones.

The kinetic parameters of TpdE were also determined (Table 1). The apparent *K_M_* for the reduction of diacetyl was 1.9 mM. Interestingly, the *K_M_* values for the reduction of 2,3-diketones varied in a chain-length dependent manner with *K_M_* values that increased with increasing substrate chain length. In addition, the enzyme had a slight preference for keto esters over either diketones or acetoin derivatives, and had the highest affinity (*K_M_* = 0.76 mM) for butan-3-one-2-yl butanoate. Moreover, the specificity constant (*k*_cat_/*K_M_* = 4,472 s^−1^ M^−1^) value for this substrate was 1.4-fold higher than that for diacetyl. The specificity constants determined for TpdE were of the same order of magnitude as those of other enzymes active towards vicinal diketones including aldo–keto reductase AKR3C1 from *Saccharomyces cerevisiae*, oxidoreductase GOX0313 from *Gluconobacter oxydans* and acetoin(diacetyl) reductase from *Rhodococcus erythropolis* WZ010 (Calam et al., 2013; Schweiger et al., 2013; Wang et al., 2014). While the bioconversion of diketones to corresponding mono- or dihydroxy alcohols, catalyzed by different alcohol dehydrogenases from various sources, have already been described (Kreit & Elalami, 2002; Edegger et al., 2006; Calam et al., 2013; Schweiger et al., 2013; Park et al., 2014), the ability of SDRs to reduce *α*-keto esters has been poorly characterized to date. Thus, TpdE has shown a novel and unique specificity, increasing our understanding of the enzymatic activity of SDRs.

**Table 1:** Kinetic parameters for reduction of carbonyls by TpdE. Parameters were determined as described in ‘Materials and Methods’.

Substrate	*K_M_*, mM	*k*_cat_, s^−1^	*k*_cat_/*K_M_*, s^−1^ ⋅ M^−1^
Diacetyl	1.9 ± 0.3	6.1 ± 0.3	3,200 ± 400
2,3-Pentanedione	0.98 ± 0.2	5.7 ± 0.4	4,000 ± 1,100
2,3-Hexanedione	2.2 ± 0.4	5.3 ± 0.2	2,400 ± 400
2,3-Heptanedione	6.9 ± 0.9	5.3 ± 0.2	760 ± 90
3,4-Hexanedione	3.3 ± 0.6	4.4 ± 0.2	1,300 ± 200
Butan-3-one-2-yl-butanoate	0.76 ± 0.1	3.8 ± 0.1	4,472 ± 410
Acetoin	5.0 ± 0.3	4.1 ± 0.3	820 ± 90
3-Hydroxy-3-methyl-2-butanone	14.6 ± 4.0	2.8 ± 0.2	190 ± 40
3-Mercapto-2-butanone	14.4 ± 4.8	3.8 ± 0.4	260 ± 60
3-Bromo-2-butanone	3.75 ± 0.7	5.2 ± 0.2	1,400 ± 200

**Figure 6:**
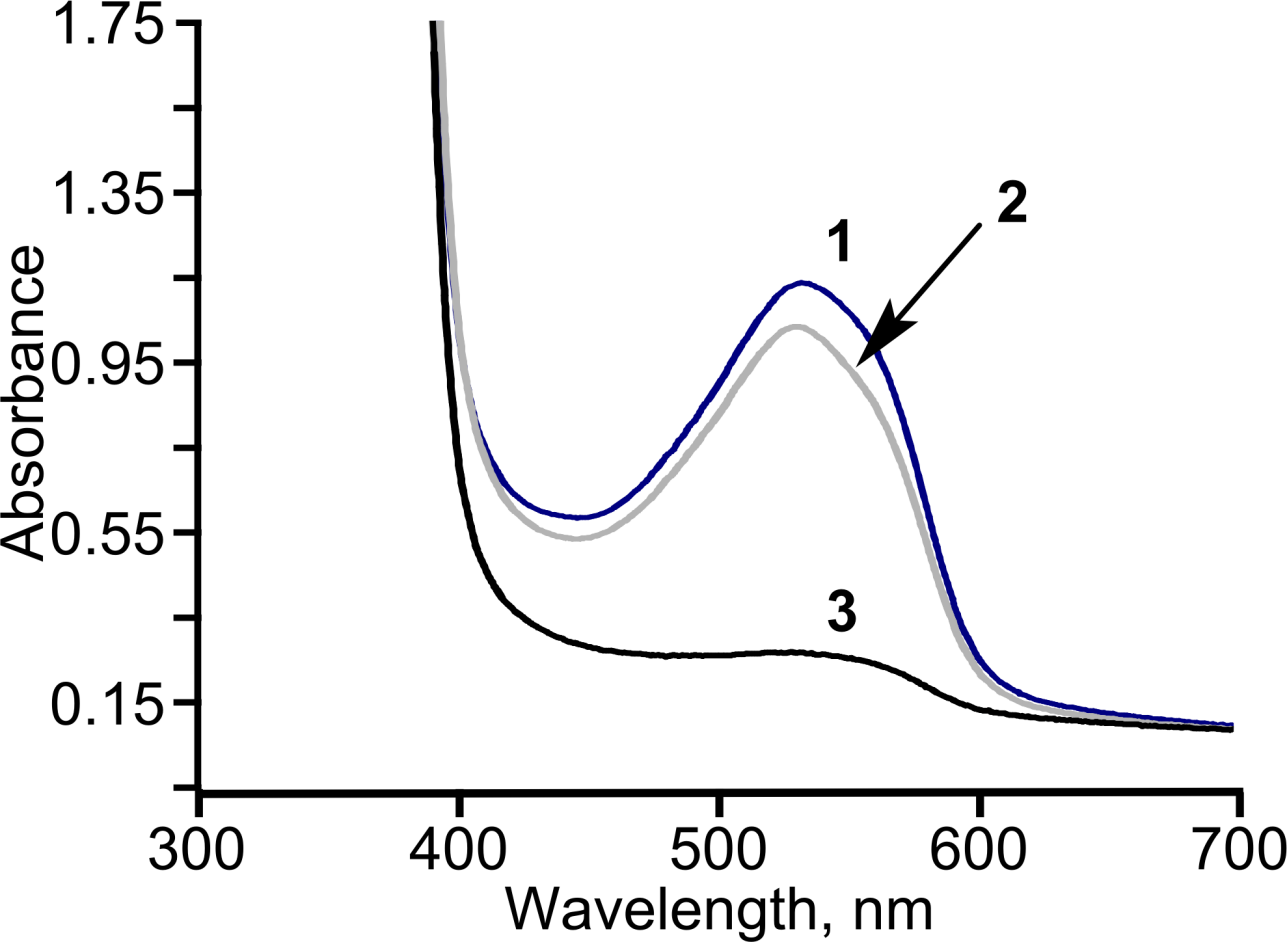
The consumption of diacetyl in *E. coli* BL21 (DE3) pTpdE cells. Diacetyl was derivatized with creatine in the presence of *α*-naphthol prior analysis. 1, initial absorption of diacetyl; 2, control sample; diacetyl was incubated without cells in 50 mM phosphate buffer, pH = 7.2, for 18 h, 3, diacetyl together with the cells was incubated for 18 h.

Since NADPH serves as a co-substrate for TpdE, a constant supply of NADPH must be ensured for a successful biocatalysis. For this reason, a whole-cell biocatalyst could be highly advantageous. Therefore, the bioconversion of diacetyl to 2,3-butanediol by *E. coli* BL21 (DE3) cells harbouring pTpdE was investigated. As shown in Fig. 6, diacetyl was almost completely consumed after 18 h of incubation. Thus, *E. coli* BL21 (DE3) pTpdE cells can act as a whole-cell biocatalyst for production of 2,3-butanediol. In parallel experiments, it was demonstrated that the TpdE expressing cells are also capable of reducing 2,3-pentanedione, 2,3-hexanedione and 2,3-heptanedione. The transformation of diketones by *E. coli* BL21 (DE3) carrying an empty pET vector was negligible.

**Figure 7:**
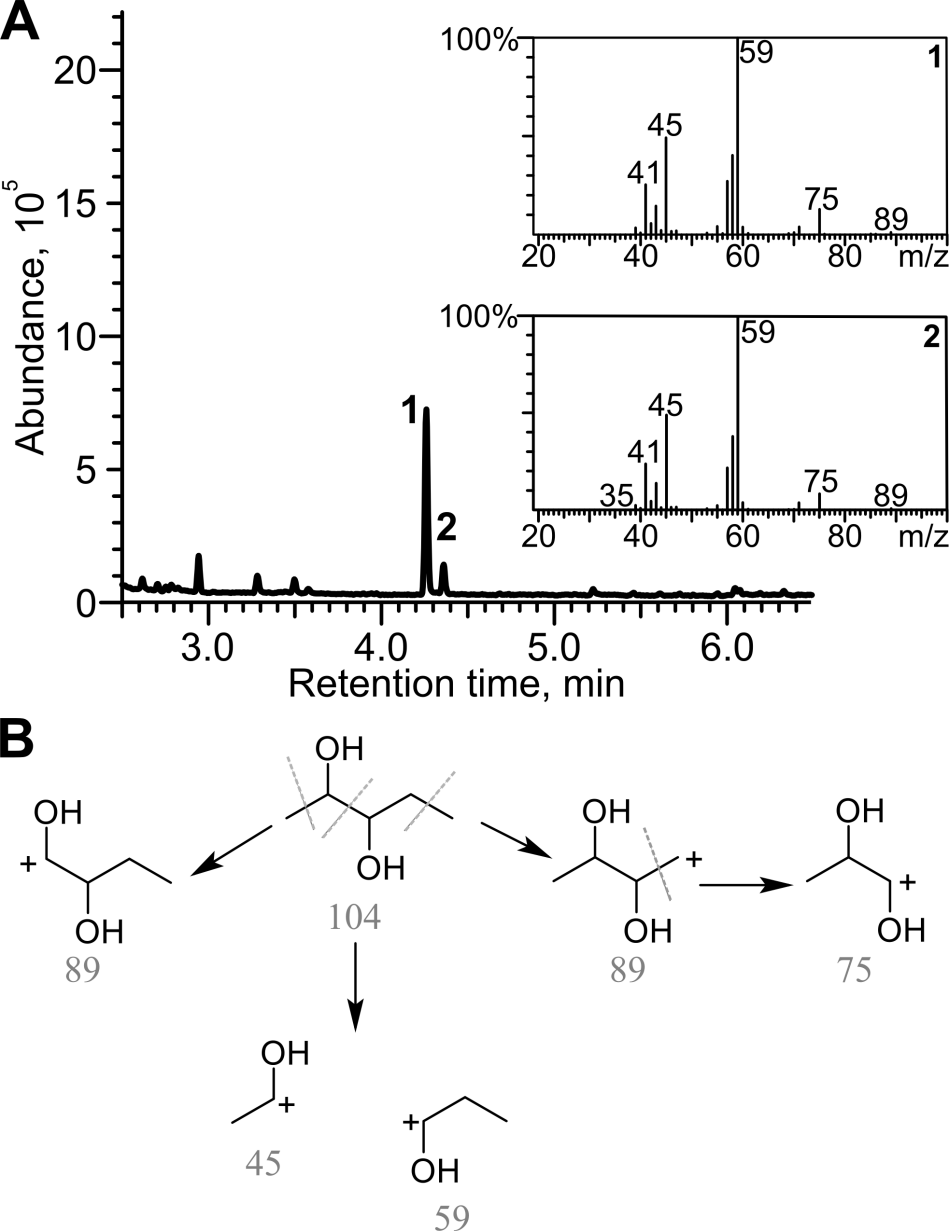
Analysis of the products of 2,3-pentanedione reduction by TpdE. (A) Chromatogram and masses of the molecule fragments are presented. Analysis performed by GC-MS. (B) The scheme of fragmentation of 2,3-pentanediol. Numbers below the fragments indicate the mass to charge ratio (*m/z*).

Diacetyl was reduced in the presence of TpdE to 2,3-butanediol, therefore a process of reduction of various 2,3- and 3,4-diketones by *E. coli* BL21 (DE3) pTpdE cells was investigated aiming to determine the reaction products. GC-MS analysis revealed that a single dominant product was formed after the reduction of 2,3-pentanedione using TpdE as a catalyst. Two main fragments of the product were formed: *m*/*z* 45 and *m*/*z* 59 (Fig. 7A), while the fragmentation of 2,3-pentanedione resulted in *m*/*z* 43 and *m*/*z* 57 (Fig. S5). The fragmentation of the product matched the predicted fragmentation pattern of 2,3-pentanediol (Fig. 7B). Thus, both keto groups of 2,3-pentanedione were reduced by TpdE. The minor product was observed in the chromatogram (Figs. 7A and 2). Since it formed the same fragments as those obtained from the major product, it could be assumed that stereoisomers of the diol were produced, and partially separated during the analysis.

2,3-Hexanedione, 3,4-hexanedione, 2,3-heptanedione, as well as the products of their biotransformation, were also analyzed by GC-MS (Fig. S5). 2,3-Hexanedione formed two major fragments: *m*/*z* 43 and *m*/*z* 71, whereas the two fragments (*m*/*z* 45 and *m*/*z* 73) of the reduction product were in agreement with the formation of 2,3-hexanediol. Symmetric substrate 3,4-hexanedione was split into two identical fragments with *m*/*z* 57, while the fragmentation pattern of the product matched that of a symmetric compound, forming a prevailing ion (*m*/*z* 59). Thus, TpdE is capable of reducing both keto groups in 3,4-hexanedione. 2,3-Heptanedione formed characteristic fragments of *m*/*z* 43 and *m*/*z* 85. In the case of 2,3-heptanedione reduction, the masses of the product fragments increased by 2 Da and were 45 Da and 87 Da respectively. Therefore, 2,3-heptanediol was identified as the product of 2,3-heptanedione reduction mediated by TpdE.

**Figure 8:**
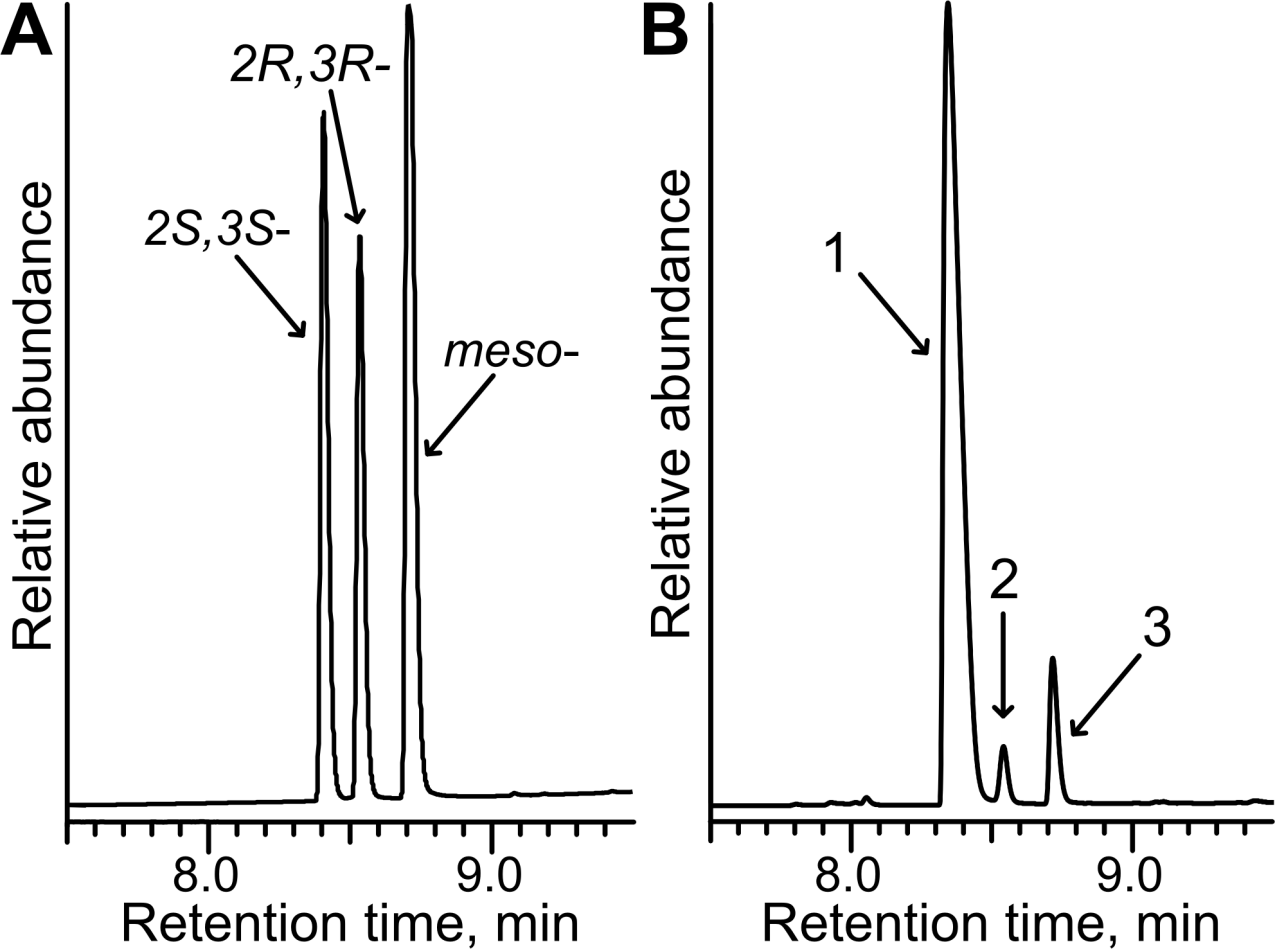
Stereoselective synthesis of 2,3-butanediol by TpdE. (A) Chromatogram of the mixture of *2R,3R-*, *2S,3S-* and *meso-*butanediol standards. (B) Chromatogram of diacetyl biotransformation mixture. Whole cells harbouring TpdE were used for the reaction. 1, 2 and 3 indicate enantiomers of 2,3-butanediol. The analysis was performed by GC-MS.

Since the reduction of 2,3- and 3,4-diketones by TpdE resulted in the formation of two chiral centers, the stereospecificity of the products was investigated. Biotransformation products of diacetyl were analyzed by GC, and compared with the chromatogram of commercial standards of potential products, 2*R*, 3*R*-, 2*S*, 3*S*- and *meso*-butanediol. The mixture of standards separated into three peaks (Fig. 8A). In the chromatogram of the diacetyl biotransformation sample, all three peaks representing 2,3-butanediols were assigned (Fig. 8B). The ratio of 2*S*, 3*S*/2*R*, 3*R*/*meso* 2,3-butanediols was 29:1:3 suggesting that diacetyl was reduced stereoselectively by TpdE. Enantio purity (89%) of *S*, *S*-enantiomer and diastereomeric excess (84% *de*) were comparable with those for (2*S*, 3*S*)-(+)-2,3-butanediol (10–99%) produced by the optimized whole-cell processes (Ui et al., 2004; Celinska & Grajek, 2009; Li et al., 2012).

As seen in Fig. 8B, the quantity of *meso*-2,3-butanediol was 3-fold higher compared to that of 2*R*, 3*R*-enantiomer. This suggests that when an intermediate product was *R*-acetoin, it was further reduced with stereopreference leading to a *meso-*alcohol. An additional experiment examining the stereopreference of the TpdE was conducted. Here, the oxidation reaction was assayed spectrophotometrically using optically pure 2,3-butanediols as substrates. Formation of the product was observed only with (2*S*, 3*S*)-(+)-2,3-butanediol as a substrate. These results revealed that TpdE catalyzes the stereoselective reduction of diketones to the corresponding diols.

**Figure 9:**
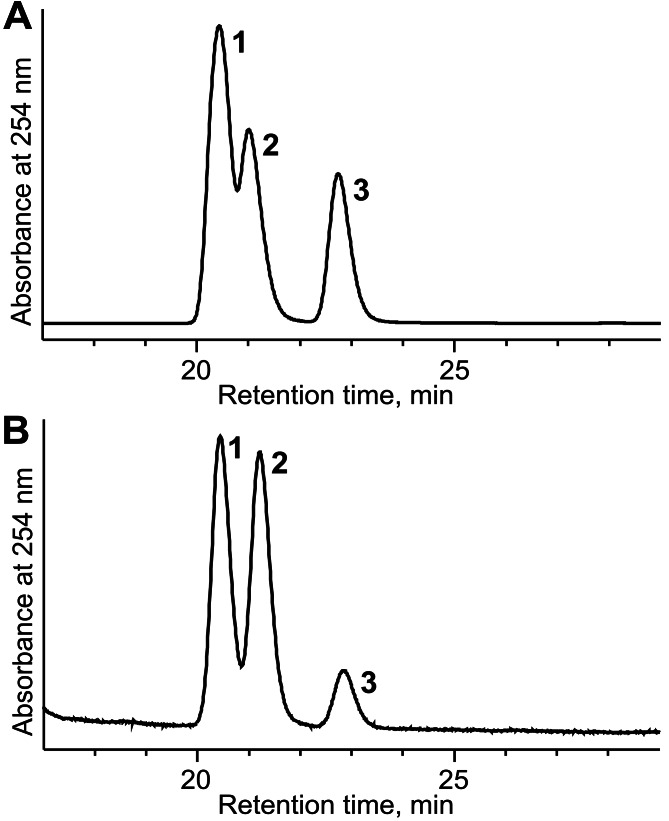
Chiral separation of 3-hydroxybutan-2-yl-nicotinate enantiomers. The enantiomers were synthesized from butan-3-one-2-yl nicotinate (A) after chemical reduction, using NaBH_4_ and (B) after bioconversion, using whole cells of *E. coli* BL21 (DE3) pTpdE. 1, mixture of *2R,3S* and *2S,3R* enantiomers; 2, *2S,3S* enantiomer; 3, *2R,3R* enantiomer.

The potential of *E. coli* BL21 (DE3) pTpdE cells for the biocatalytic asymmetric reduction of butan-3-one-2-yl nicotinate was further investigated. The overnight grown cells were transferred to phosphate buffer (10 mM, pH 7.2) containing glucose 0.25% (w/v) and butan-3-one-2-yl nicotinate 0.05% (w/v). After 20 h the bioconversion mixture was studied by HPLC-MS. The data revealed that complete reduction of the substrate occurred. This reduction produced a new chiral center, thus the enantiomeric ratio of the product was examined. Biotransformation products were analyzed by HPLC and compared to the products formed after chemical reduction. As shown in Fig. 9A, the mixture of the reduction products, obtained using NaBH_4_, migrated as three peaks: the first peak corresponded to the mixture of 2*R*, 3*S* and 2*S*, 3*R*, the second incompletely separated peak belonged to 2*S*, 3*S* enantiomer and the third fully separated peak corresponded to 2*R*, 3*R* enantiomer. The ratio of the peak area was 3:1 (unseparated peaks were calculated as one). The same peaks were detected after biotransformation of racemic butan-3-one-2-yl nicotinate using *E. coli* BL21(DE3) pTpdE cells (Fig. 9B). Nevertheless, the ratio of the peaks differed and corresponded to 10:1 and only small amounts of *R*, *R* enantiomer were formed, indicating that butan-3-one-2-yl nicotinate was reduced stereoselectively by *E. coli* BL21(DE3) pTpdE cells.

The reduction of chiral *α*-ketoesters by biotransformation is preferred over chemical techniques due to mild reaction conditions and avoids the toxic catalysts used in organic synthesis. Consequently, TpdE is a potential biocatalyst for the production of industrially important derivatives of *α*-hydroxy esters that are generally difficult to synthesize chemically. Thus, optimization of *E. coli* BL21(DE3) pTpdE as a whole-cell biocatalyst and the analysis of TpdE regioselectivity and stereospecificity warrant further study.

## Conclusions

The NADPH-dependent protein TpdE from *Rhodococcus jostii* TMP1 is a new member of the “classical” short-chain dehydrogenase/reductase superfamily. This enzyme catalyzes a reduction of various 2,3- and 3,4-diketones to corresponding diols. Furthermore, TpdE exhibits high catalytic activity for the reduction of diverse keto esters. Since TpdE catalyzes a stereoselective reduction, it can be successfully used for the synthesis of chiral alcohols by asymmetric ketone reduction both *in vivo* and *in vitro*.

## Supplemental Information

10.7717/peerj.1387/supp-1 Figure S1 Purification of the recombinant TpdE protein TpdE was purified from the cell-free extracts by affinity chromatography using Ni^2+^-chelating column. SDS-PAGE gel was stained with Coomassie Blue. M lane, protein molecular mass marker (kDa); 1 lane, TpdE (∼30 kDa) after purification.

10.7717/peerj.1387/supp-2 Figure S2 The effect of pH on TpdE activity The activity was investigated within a range from 4.0 to 9.5 at 30 °C. The activity was assayed spectrophotometrically measuring as described in Materials and Methods. In all reactions diacetyl was used as the second substrate. The concentration of the buffers was 50 mM.

10.7717/peerj.1387/supp-3 Figure S3 The effect of metal ions on TpdE The activity was assayed in the presence of various metal ions spectrophotometrically measuring as described in Materials and Methods. Experiments were performed in triplicate and activity without metal ions was set as 100%.

10.7717/peerj.1387/supp-4 Figure S4 The effect of temperature on TpdE activity and stability (A) The activity was measured at different temperatures in phosphate buffer (50 mM, pH 7.2) containing 0.2 mM NADPH and 10 mM diacetyl. (B) For thermostability, the enzyme solution was kept at different temperatures for 10 min in phosphate buffer and then immediately cooled on ice. The residual activity was measured at 30 °C as described in Materials and Methods.

10.7717/peerj.1387/supp-5 Figure S5 GC-MS spectra for substrates and products of the reactions catalyzed by TpdE Biotransformations of (A) 2,3-pentanedione, (B) 2,3-hexanedione, (C) 3,4-hexanedione and (D) 2,3-heptanedione are presented.

10.7717/peerj.1387/supp-6 Supplemental Information 1 Raw Data

## References

[ref-1] Bennet GN, San KY (2001). Microbial formation, biotechnological production and applications of 1,2-propanediol. Applied Microbiology and Biotechnology.

[ref-2] Calam E, Porté S, Fernández MR, Farrés J, Parés X, Biosca JA (2013). Biocatalytic production of alpha-hydroxy ketones and vicinal diols by yeast and human aldo–keto reductases. Chemico-Biological Interactions.

[ref-3] Celinska E, Grajek W (2009). Biotechnological production of 2,3-butanediol–current state and prospects. Biotechnology Advances.

[ref-4] Chen Y, Chen C, Wu X (2012). Dicarbonyl reduction by single enzyme for the preparation of chiral diols. Chemical Society Reviews.

[ref-5] Chen C, Wei D, Shi J, Wang M, Hao J (2014). Mechanism of 2,3-butanediol stereoisomer formation in *Klebsiella pneumonia*. Applied Microbiology and Biotechnology.

[ref-6] Edegger K, Stampfer W, Seisser B, Faber K, Mayer SF, Oehrlein R, Hafner A, Kroutil W (2006). Regio- and stereoselective reduction of diketones and oxidation of diols by biocatalytic hydrogen transfer. European Journal of Organic Chemistry.

[ref-7] Gao J, Yang HH, Feng XH, Li S, Xu H (2013). A 2,3-butanediol dehydrogenase from *Paenibacillus polymyxa* ZJ-9 for mainly producing *R*, *R*-2,3-butanediol: purification, characterization and cloning. Journal of Basic Microbiology.

[ref-8] Kreit J, Elalami A (2002). Substrate characterization of a NAD-dependent secondary alcohol dehydrogenase from *Rhodococcus* sp. GK1 (CIP 105335). Journal of Molecular Catalysis B: Enzymatic.

[ref-9] Kutanovas S, Stankeviciute J, Urbelis G, Tauraite D, Rutkiene R, Meskys R (2013). Identification and characterization of a tetramethylpyrazine catabolic pathway in *Rhodococcus jostii* TMP1. Applied and Environmental Microbiology.

[ref-10] Li L, Wang Y, Zhang L, Ma C, Wang A, Tao F, Xu P (2012). Biocatalytic production of (2S,3S)-2,3-butanediol from diacetyl using whole cells of engineered *Escherichia coli*. Bioresource Technology.

[ref-11] Liu R, Berglund P, Hogberg H-E (2005). Preparation of the four stereoisomers of 3-bromo-2-butanol or their acetates via lipase-catalysed resolutions of the racemates derived from dl- or meso-2,3-butanediol. Tetrahedron: Asymmetry.

[ref-12] Neises B, Steglich W (1978). Simple method for the esterification of carboxylic acids. Angewandte Chemie International Edition.

[ref-13] Oppermann U, Filling C, Hult M, Shafqat N, Wu X, Lindh M, Shafqat J, Nordling E, Kallberg Y, Persson B, Jornvall H (2003). Short-chain dehydrogenases/reductases (SDR): the 2002 update. Chemico-Biological Interactions.

[ref-14] Park JM, Hong WK, Lee SM, Heo SY, Jung YR, Kang IY, Oh BR, Seo JW, Kim CH (2014). Identification and characterization of a short-chain acyl dehydrogenase from *Klebsiella pneumoniae* and its application for high-level production of L-2,3-butanediol. Journal of Industrial Microbiology and Biotechnology.

[ref-15] Schweiger P, Gross H, Zeiser J, Deppenmeier U (2013). Asymmetric reduction of diketones by two *Gluconobacter oxydans* oxidoreductases. Applied Microbiology and Biotechnology.

[ref-16] Tamura K, Stecher G, Peterson D, Filipski A, Kumar S (2013). MEGA6: Molecular Evolutionary Genetics Analysis Version 6.0. Molecular Biology and Evolution.

[ref-17] Ui S, Takusagawa Y, Sato T, Ohtsuki T, Mimura A, Ohkuma M, Kudo T (2004). Production of L-2,3-butanediol by a new pathway constructed in *Escherichia coli*. Letters in Applied Microbiology.

[ref-18] Wang Z, Song Q, Yu M, Wang Y, Xiong B, Zhang Y, Zheng J, Ying X (2014). Characterization of a stereospecific acetoin(diacetyl) reductase from *Rhodococcus erythropolis* WZ010 and its application for the synthesis of (2S,3S)-2,3-butanediol. Applied Microbiology and Biotechnology.

[ref-19] Xiao Z, Lv C, Gao C, Qin J, Ma C, Liu Z, Liu P, Li L, Xu P (2010). A novel whole-cell biocatalyst with NAD^+^ regeneration for production of chiral chemicals. PLoS ONE.

[ref-20] Yan Y, Lee C, Liao J (2009). Enantioselective synthesis of pure (*R*, *R*)-2,3-butanediol in *Escherichia coli* with stereospecific secondary alcohol dehydrogenases. Organic & Biomolecular Chemistry.

[ref-21] Yang C, Ying X, Yu M, Zhang Y, Xiong B, Song Q, Wang Z (2012). Towards the discovery of alcohol dehydrogenases: NAD(P)H fluorescence-based screening and characterization of the newly isolated *Rhodococcus erythropolis* WZ010 in the preparation of chiral aryl secondary alcohols. Journal of Industrial Microbiology and Biotechnology.

[ref-22] Yu B, Sun J, Bommareddy RR, Song L, Zeng AP (2011). Novel (2*R*, 3*R*)-2,3-butanediol dehydrogenase from potential industrial strain *Paenibacillus polymyxa* ATCC 12321. Applied and Environmental Microbiology.

[ref-23] Zhang L, Xu Q, Zhan S, Li Y, Lin H, Sun S, Sha L, Hu K, Guan X, Shen Y (2014). A new NAD(H)-dependent *meso*-2,3-butanediol dehydrogenase from an industrially potential strain *Serratia marcescens* H30. Applied Microbiology and Biotechnology.

[ref-24] Zhu D, Yang Y, Hua L (2006). Stereoselective enzymatic synthesis of chiral alcohols with the use of a carbonyl reductase from *Candida magnoliae* with anti-Prelog enantioselectivity. Journal of Organic Chemistry.

