# Supplementary figures and images for: Ketoreductase TpdE from Rhodococcus jostii TMP1: characterization and application in the synthesis of chiral alcohols
Source: PeerJ. 2015 Nov 10;3:e1387. doi: 10.7717/peerj.1387 (PMC4647570; doi:10.7717/peerj.1387)

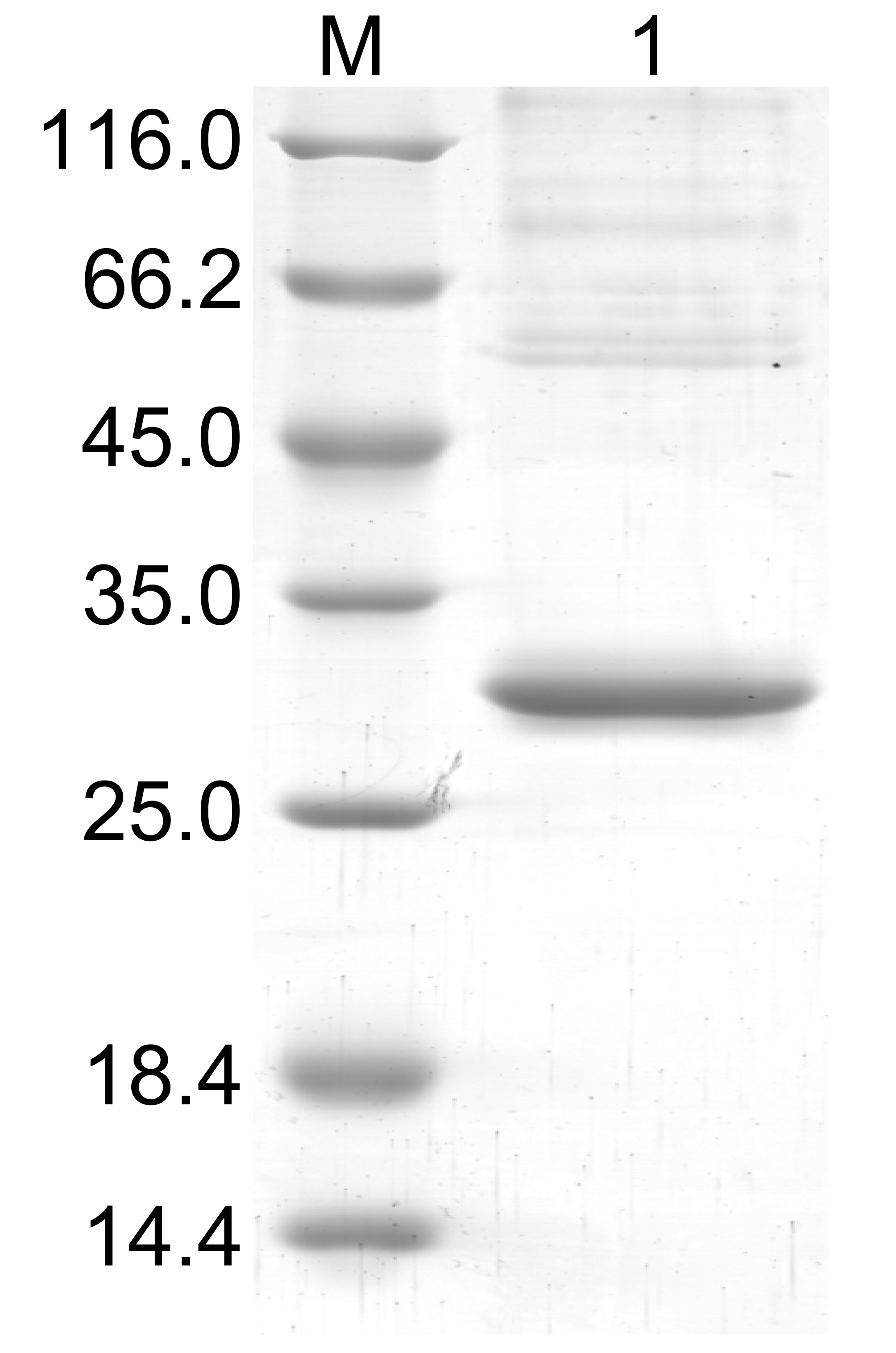

Supplement: Figure S1 — TpdE was purified from the cell-free extracts by affinity chromatography using Ni2+-chelating column. SDS-PAGE gel was stained with Coomassie Blue. M lane, protein molecular mass marker (kDa); 1 lane, TpdE (∼30 kDa) after purification. [file peerj-03-1387-s001.png]

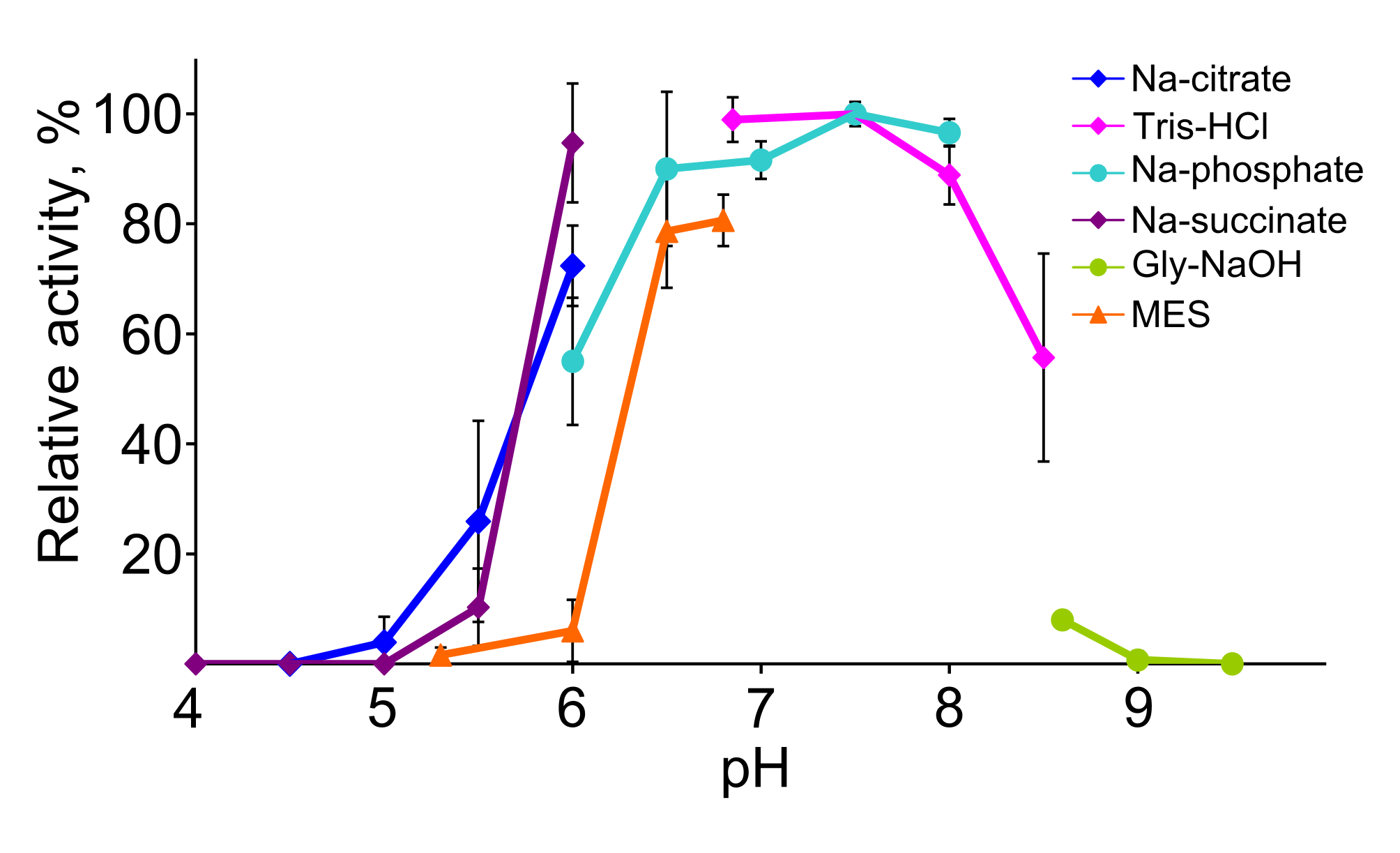

Supplement: Figure S2 — The activity was investigated within a range from 4.0 to 9.5 at 30 °C. The activity was assayed spectrophotometrically measuring as described in Materials and Methods. In all reactions diacetyl was used as the second substrate. The concentration of the buffers was 50 mM. [file peerj-03-1387-s002.png]

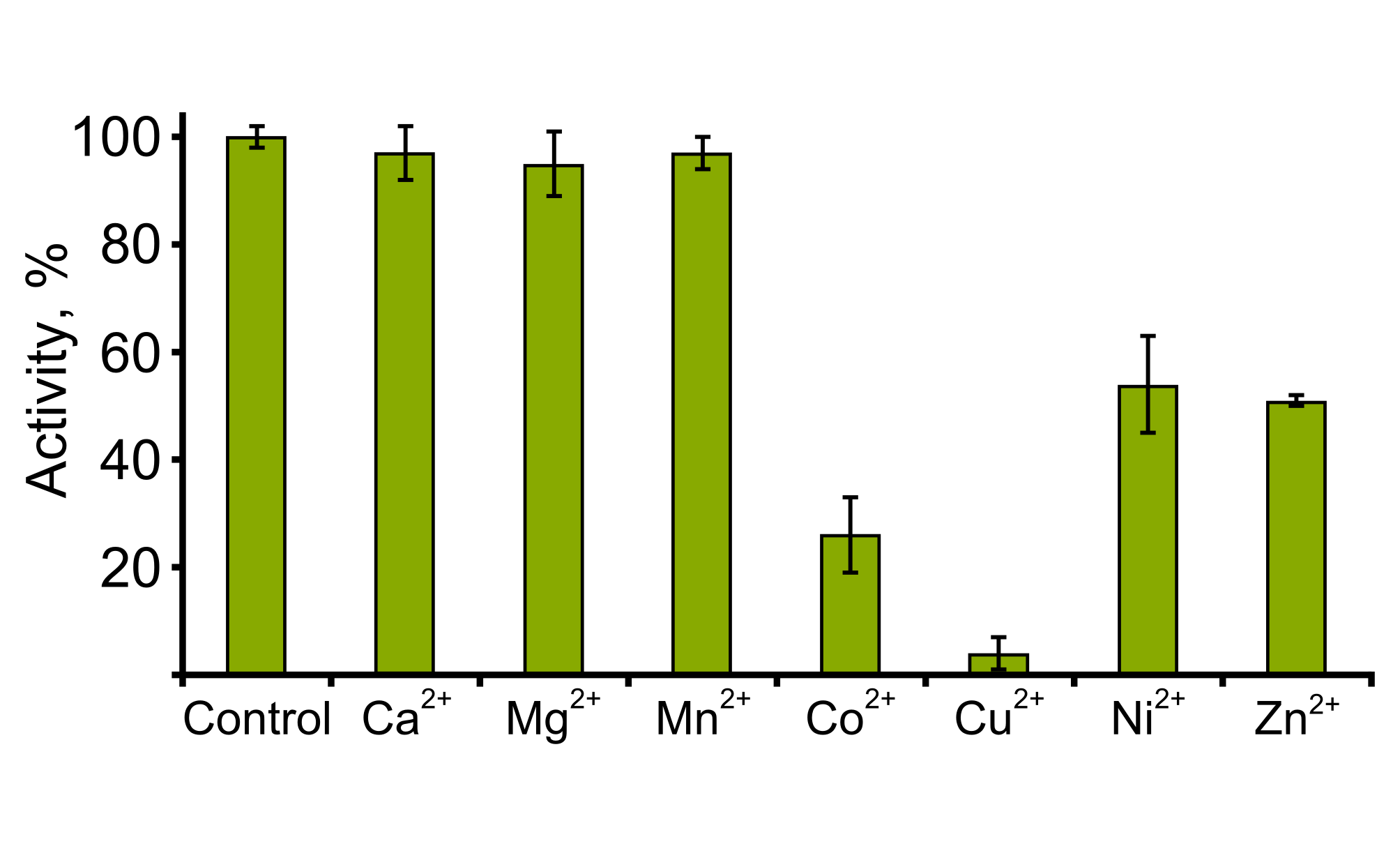

Supplement: Figure S3 — The activity was assayed in the presence of various metal ions spectrophotometrically measuring as described in Materials and Methods. Experiments were performed in triplicate and activity without metal ions was set as 100%. [file peerj-03-1387-s003.png]

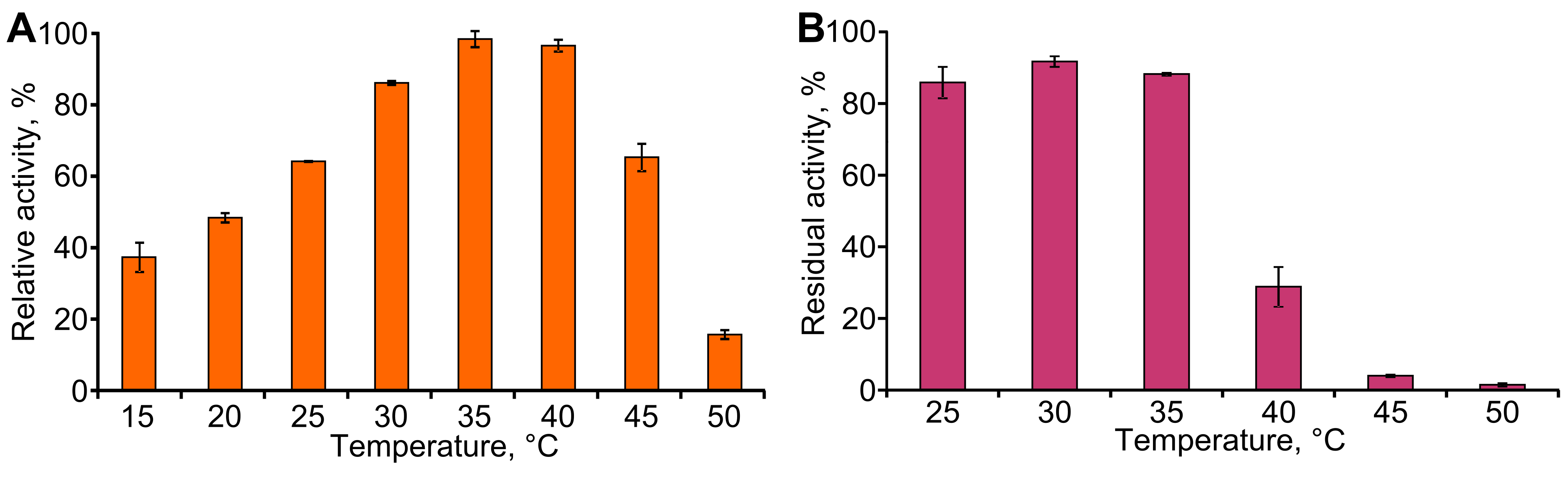

Supplement: Figure S4 — (A) The activity was measured at different temperatures in phosphate buffer (50 mM, pH 7.2) containing 0.2 mM NADPH and 10 mM diacetyl. (B) For thermostability, the enzyme solution was kept at different temperatures for 10 min in phosphate buffer and then immediately cooled on ice. The residual activity was measured at 30 °C as described in Materials and Methods. [file peerj-03-1387-s004.png]

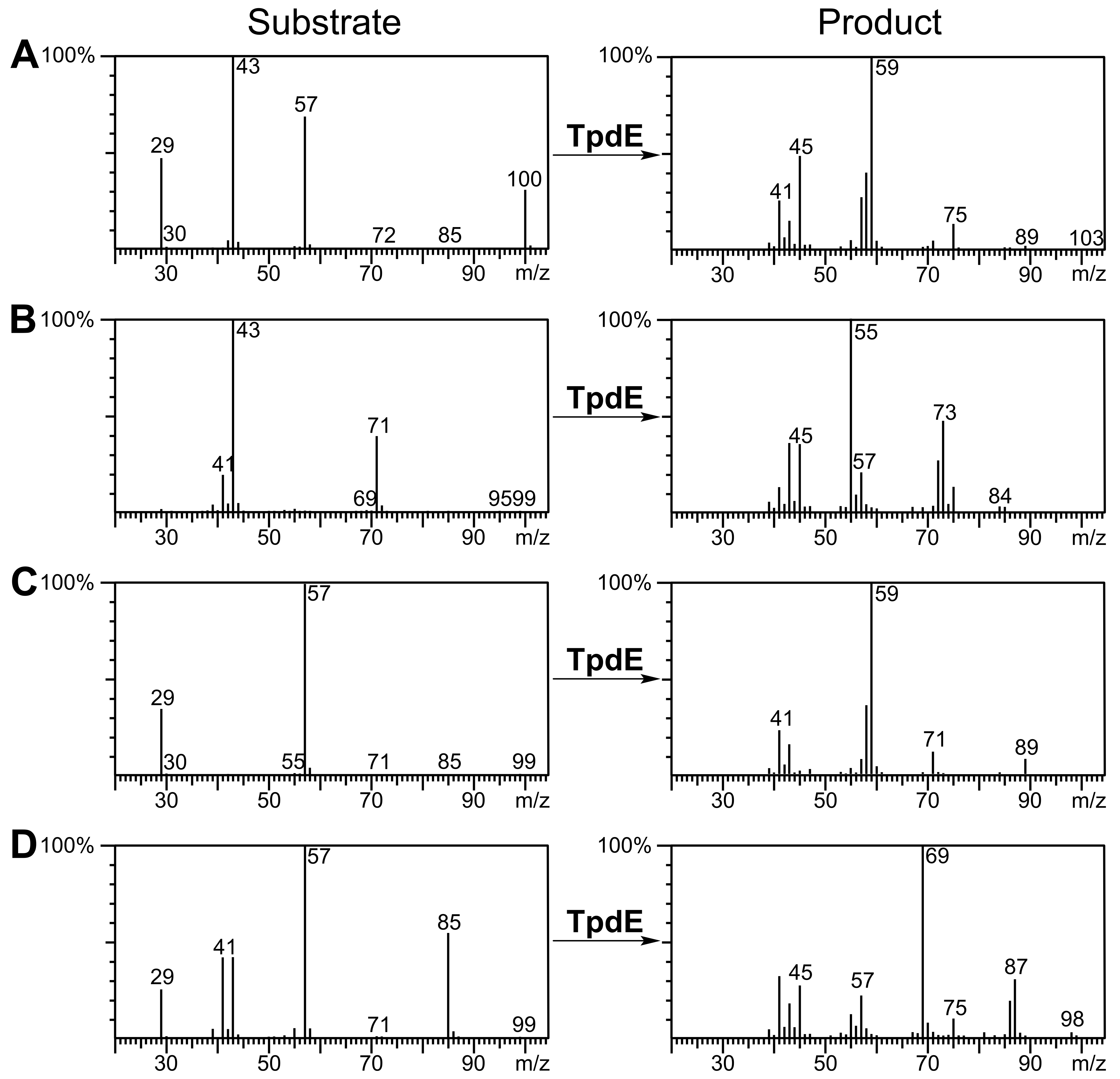

Supplement: Figure S5 — Biotransformations of (A) 2,3-pentanedione, (B) 2,3-hexanedione, (C) 3,4-hexanedione and (D) 2,3-heptanedione are presented. [file peerj-03-1387-s005.png]

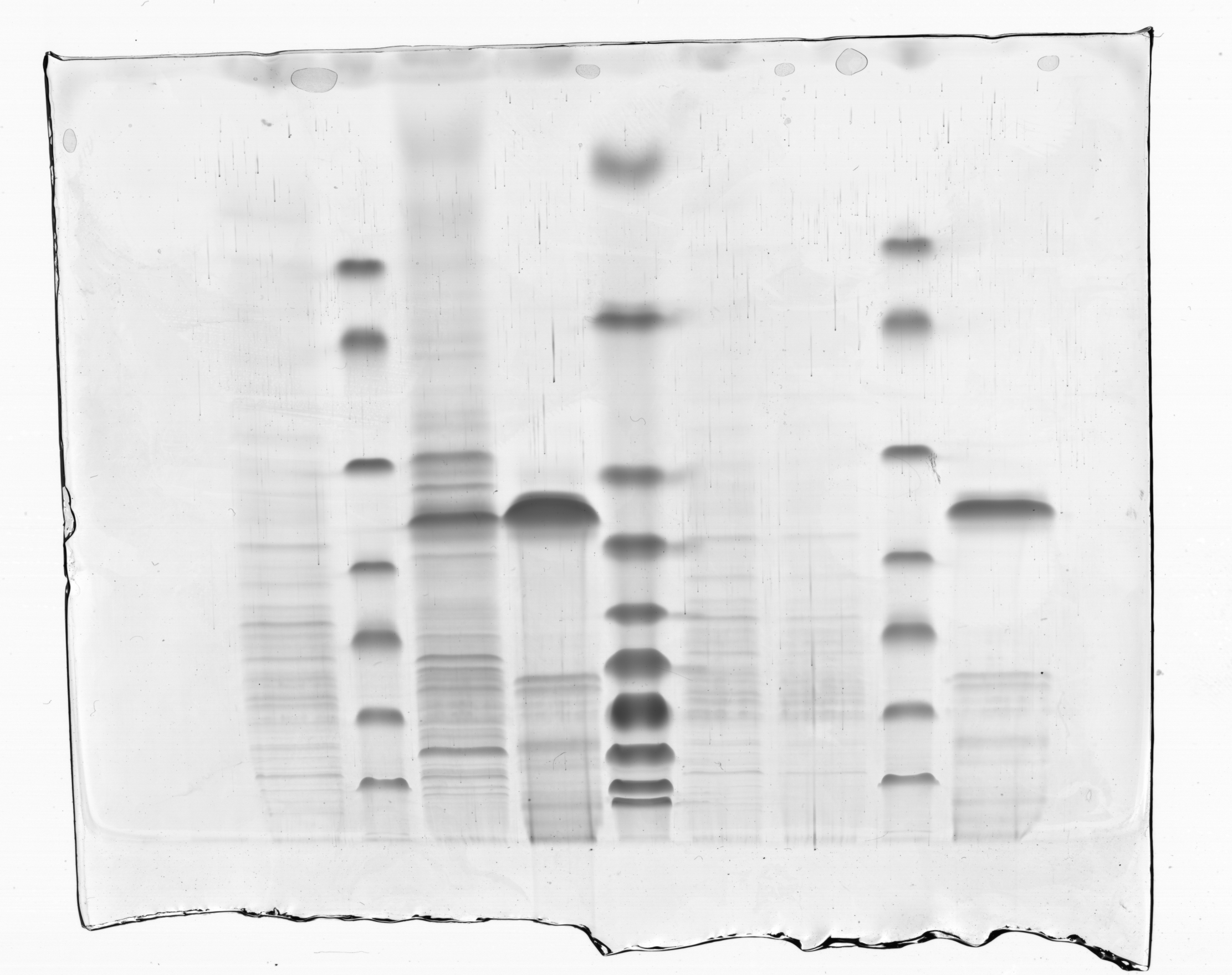

Supplement: Supplemental Information 1 [file peerj-03-1387-s006.zip › Raw data/TpdE gel electrophoresis.tif]
