# Supplementary material for: Ketoreductase TpdE from Rhodococcus jostii TMP1: characterization and application in the synthesis of chiral alcohols
Source: PeerJ. 2015 Nov 10;3:e1387. doi: 10.7717/peerj.1387 (PMC4647570; doi:10.7717/peerj.1387)
Supplement: Supplemental Information 1 [file peerj-03-1387-s006.zip › Raw data/2,3-butanediols standards GC.pdf]

Analysis Date & Time : 8/31/2015 4:52:41 PM
User Name : Admin
Vial# : 1
Sample Name : Mix
Sample ID :
Sample Type : Unknown
Injection Volume : 0,20
ISTD Amount :

Data Name : C:\GCsolution\Ritos\R_S_mezo.gcd
Method Name : C:\GCsolution\Ritos\Butandiolis_RS.gcm
[Description]
R-2,3-Butanediol 9mkl/1ml
S-2,3-Butanediol 9mkl/1ml
mezo_2,3-Butanediol 15,1mg/1ml
Ir tokiu tirpalu misinys 1:1:1
Mtd Butandiolis 0,2mkl

Mtd Butandiolis 0,2mkl

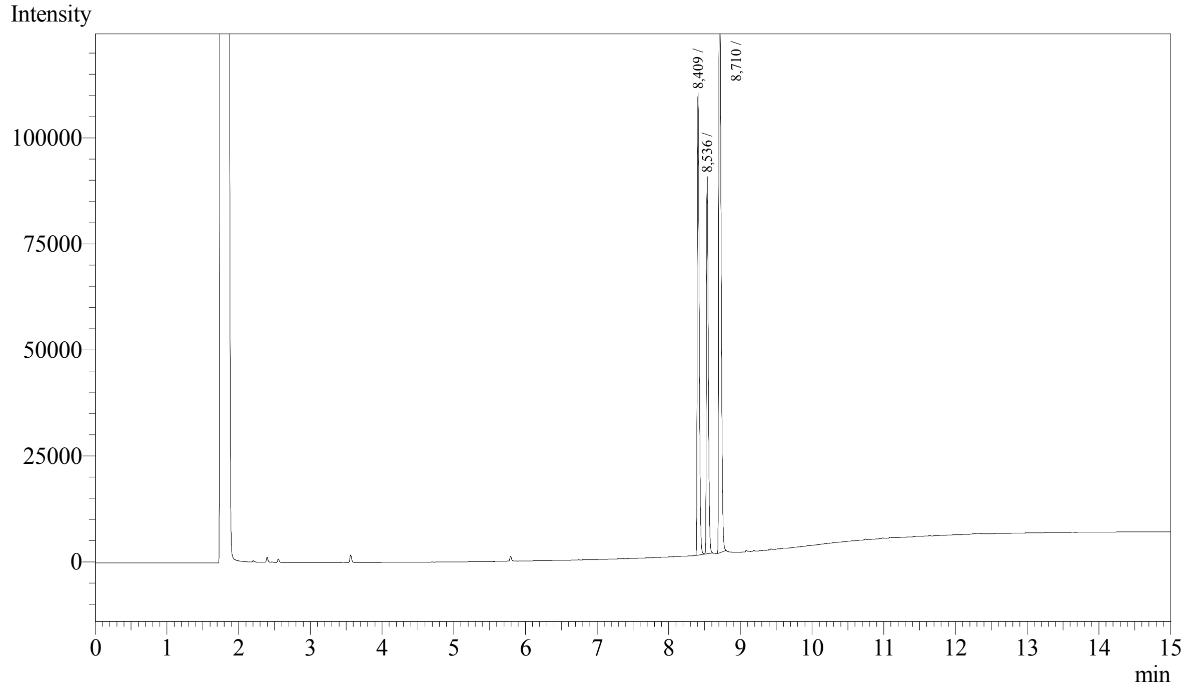

Peak#	Ret.Time	Area	Height	Conc.	Unit	Mark	ID#	Cmpd Name
1	8,409	206461	108470	0,000				
2	8,536	163712	87090	0,000		V		
3	8,710	314508	143350	0,000				
Total		684681	338910					
