# Supplementary material for: Ketoreductase TpdE from Rhodococcus jostii TMP1: characterization and application in the synthesis of chiral alcohols
Source: PeerJ. 2015 Nov 10;3:e1387. doi: 10.7717/peerj.1387 (PMC4647570; doi:10.7717/peerj.1387)
Supplement: Supplemental Information 1 [file peerj-03-1387-s006.zip › Raw data/3-Hydroxybutan-2-yl nicotinate produced by TpdE chiral.PDF]

SHIMADZU LabSolutions

Analysis Report

<Sample Information>

Sample Name	:		
Sample ID	:		
Data Filename	: Butan-2-olio-3-ilnikotinatas red TpdE chiral.lcd		
Method Filename	: Chiral izo heksanas izopropanolis.lcm		
Batch Filename	:		
Vial #	: 1-29	Sample Type	: Unknown
Injection Volume	: 0.5 uL		
Date Acquired	: 3/5/2014 4:30:16 PM	Acquired by	: System Administrator
Date Processed	: 4/1/2014 1:19:35 PM	Processed by	: System Administrator

<Chromatogram>

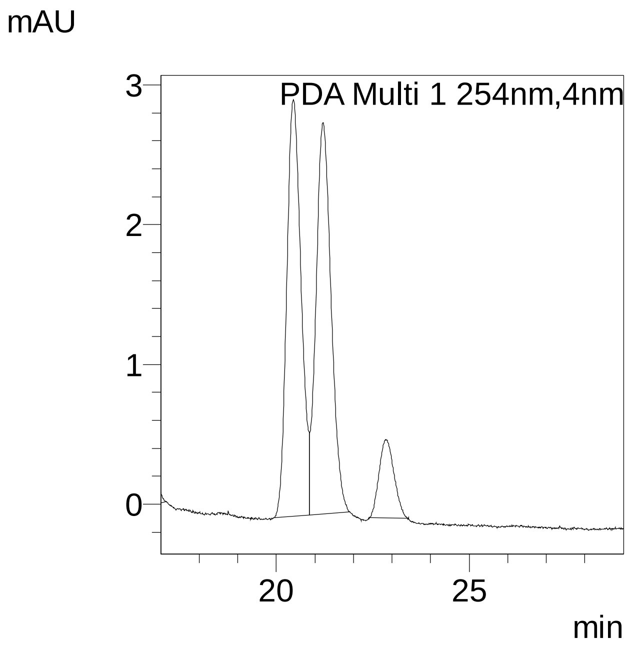

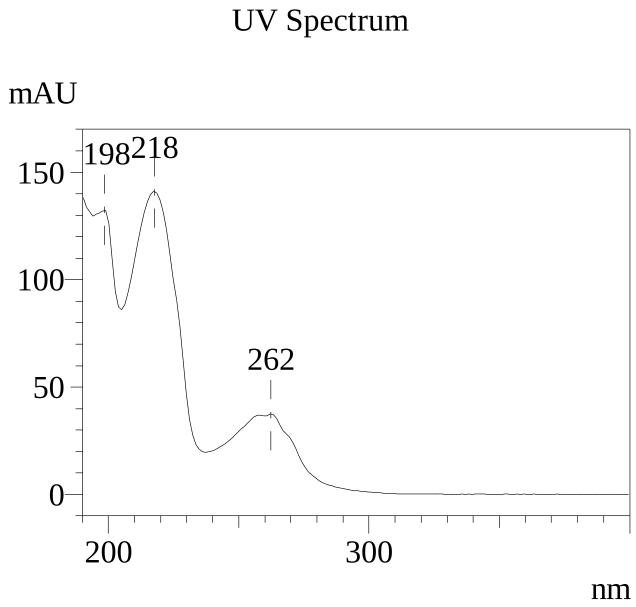

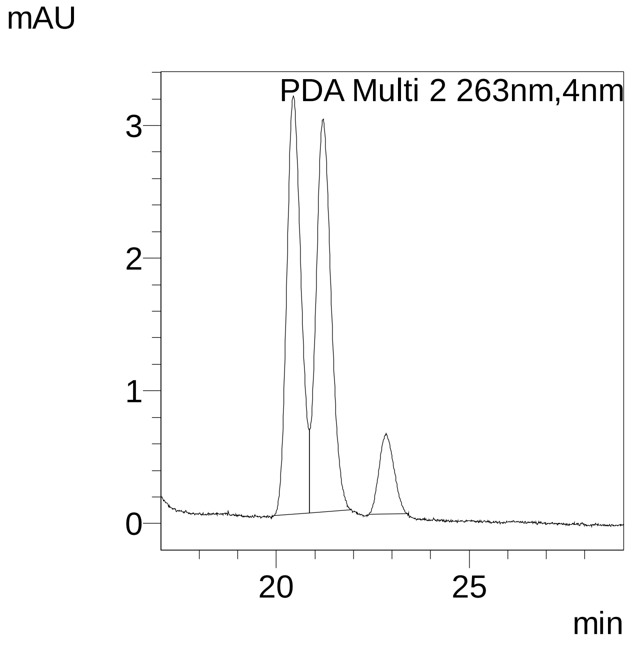
mAU
PDA Multi 2 263nm,4nm
3
2
1
0
20
25
min
