# Supplementary material for: Ketoreductase TpdE from Rhodococcus jostii TMP1: characterization and application in the synthesis of chiral alcohols
Source: PeerJ. 2015 Nov 10;3:e1387. doi: 10.7717/peerj.1387 (PMC4647570; doi:10.7717/peerj.1387)

SHIMADZU LabSolutions

Analysis Report

<Sample Information>

Sample Name	:
Sample ID	:
Data Filename	: Butan-2-olio-3-ilnikotinatas red chemiskai chiral.lcd
Method Filename	: Chiral izo heksanas izopropanolis.lcm
Batch Filename	:
Vial #	: 1-28
Sample Type	: Unknown
Injection Volume	: 0.5 uL
Date Acquired	: 3/5/2014 5:11:26 PM
Acquired by	: System Administrator
Date Processed	: 3/20/2014 7:18:41 PM
Processed by	: System Administrator

<Chromatogram>

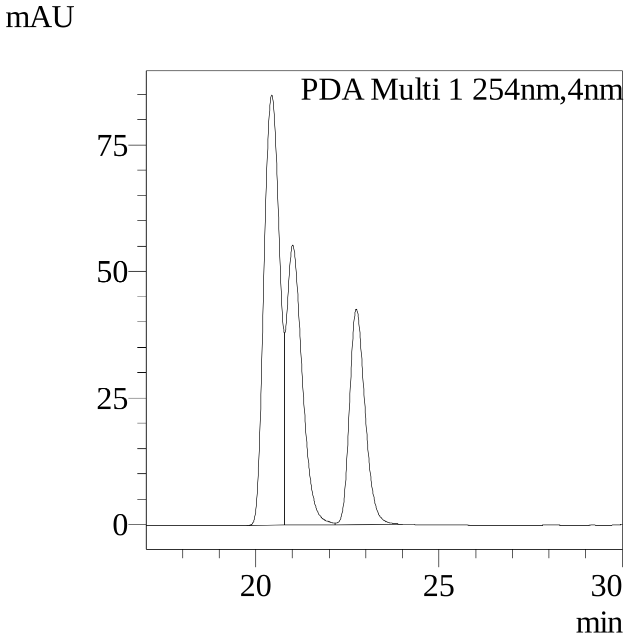

UV Spectrum

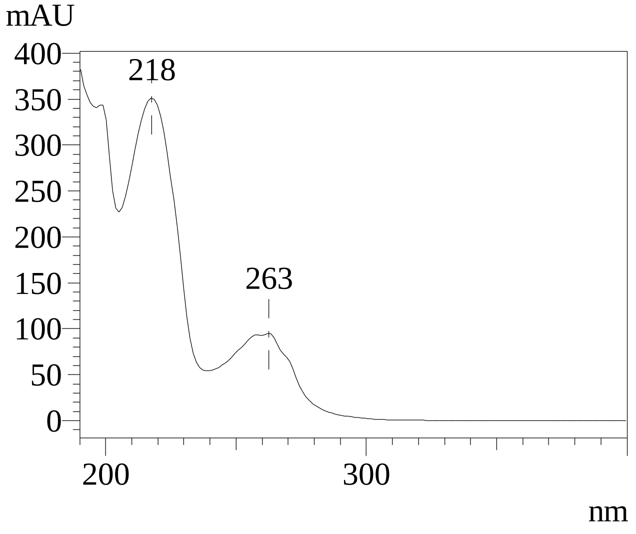

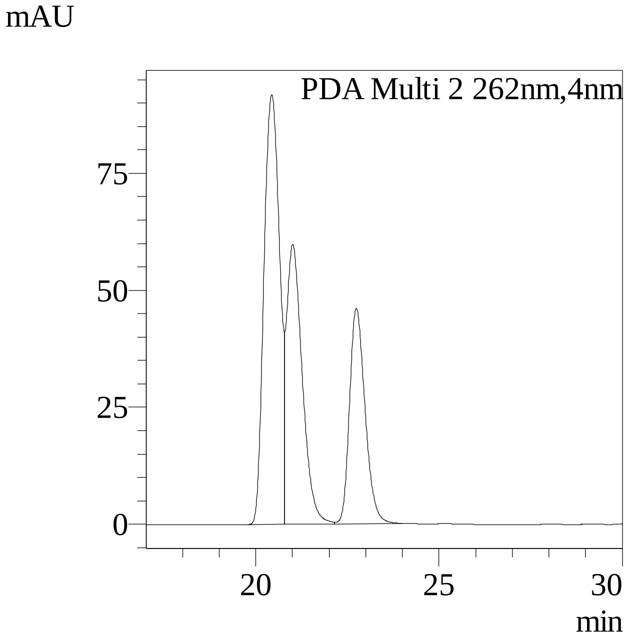

Supplement: Supplemental Information 1 [file peerj-03-1387-s006.zip › Raw data/3-Hydroxybutan-2-yl nicotinate produced NaBH4 chiral.PDF]
