# Supplementary material for: Ketoreductase TpdE from Rhodococcus jostii TMP1: characterization and application in the synthesis of chiral alcohols
Source: PeerJ. 2015 Nov 10;3:e1387. doi: 10.7717/peerj.1387 (PMC4647570; doi:10.7717/peerj.1387)
Supplement: Supplemental Information 1 [file peerj-03-1387-s006.zip › Raw data/diacetyl bioconversion GC.pdf]

Analysis Date & Time	: 9/8/2015 4:52:20 PM
User Name	: Admin
Vial#	: 3
Sample Name	: N3_Jonitos
Sample ID	:
Sample Type	: Unknown
Injection Volume	: 1,00
ISTD Amount	:

Data Name	: C:\GCsolution\Ritos\N3_Jonitos_1mkl.gcd
Method Name	: C:\GCsolution\Ritos\Butandiolis_RS.gcm

[Description]
N3 Jonitos
Mtd Butandiolis 1mkl

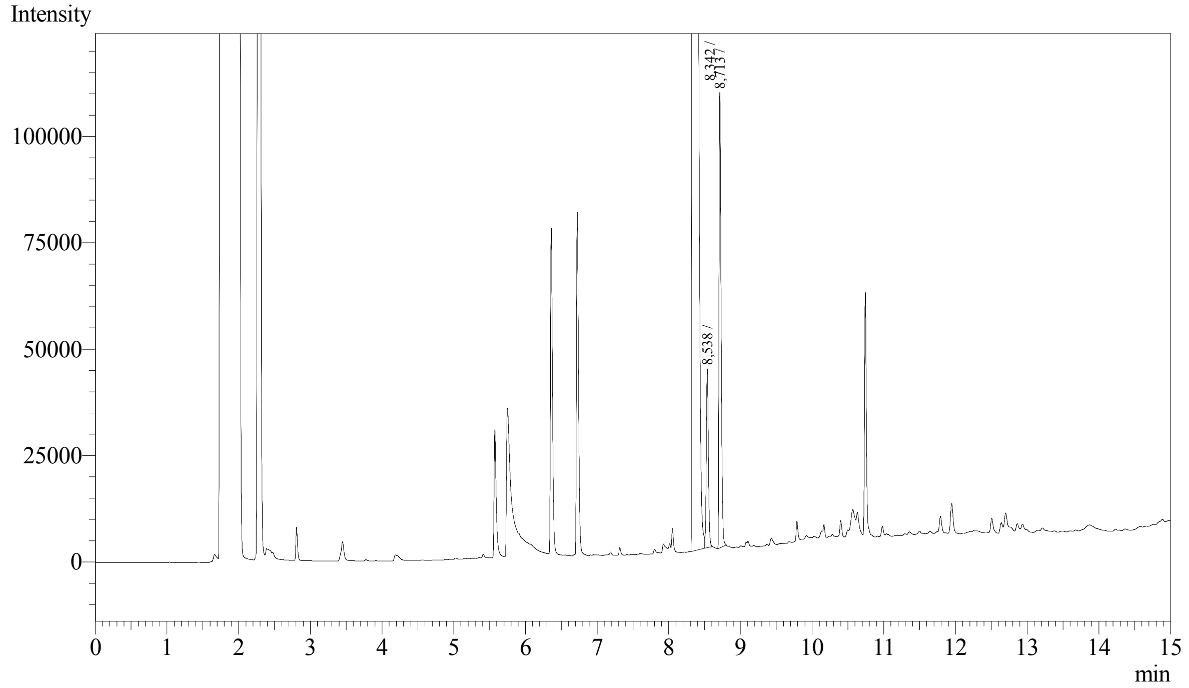

Peak#	Ret.Time	Area	Height	Conc.	Unit	Mark	ID#	Cmpd Name
1	8,342	2450810	587772	89,106				
2	8,538	85348	41696	3,103		V		
3	8,713	214290	106132	7,791				
Total		2750448	735600					
