# Supplementary material for: Ketoreductase TpdE from Rhodococcus jostii TMP1: characterization and application in the synthesis of chiral alcohols
Source: PeerJ. 2015 Nov 10;3:e1387. doi: 10.7717/peerj.1387 (PMC4647570; doi:10.7717/peerj.1387)

Sample Information

Analyzed by : Romualdas
Analyzed : 9/24/2012 1:29:45 PM
Sample Name : JoS_2
Vial # : 2
Injection Volume : 0.30
Data File : C:\GCMSsolution\Data\Project1\20120924_JoS_2.QGD
Method File : C:\GCMSsolution\Data\Project1\tirpikliai_DCM.35_Col_1701.qgm
Tuning File : C:\GCMSsolution\System\Tune1\20120910.qgt
$EndIf$Modified by : Romualdas
Modified : 9/24/2012 1:43:21 PM
Aprasymas: Jonitos_5_mix

Chromatogram JoS_2 C:\GCMSsolution\Data\Project1\20120924_JoS_2.QGD

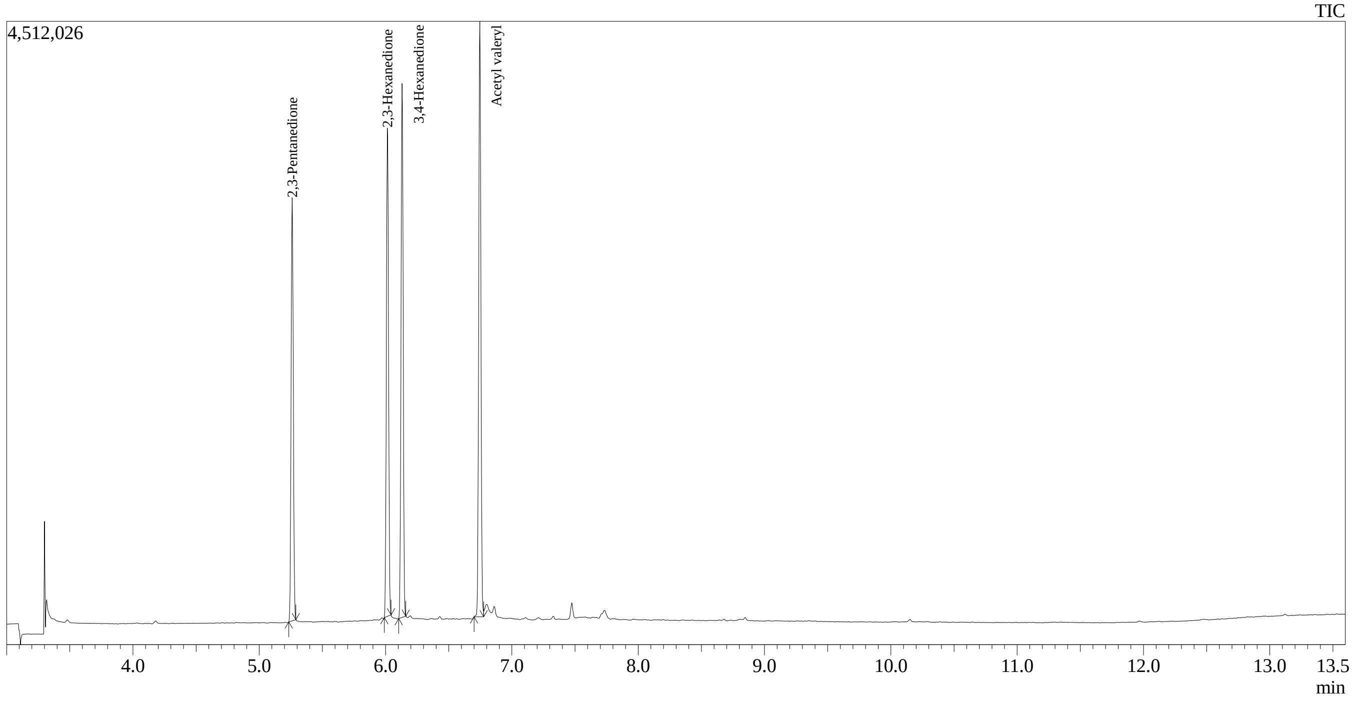

Peak Report TIC

Peak#	R.Time	Area	Area%	Mark	Name
1	5.261	3753252	20.88	MI	2,3-Pentanedione
2	6.015	4320273	24.04	MI	2,3-Hexanedione
3	6.131	4621792	25.72	MI	3,4-Hexanedione
4	6.746	5277342	29.36	MI	Acetyl valeryl
		17972659	100.00		

Library

<< Target >>
Line#:1   R.Time:5.260(Scan#:679) MassPeaks:147
RawMode:Averaged 5.257-5.263(678-680) BasePeak:43.00(1093167)
BG Mode:Calc. from Peak Group 1 - Event 1

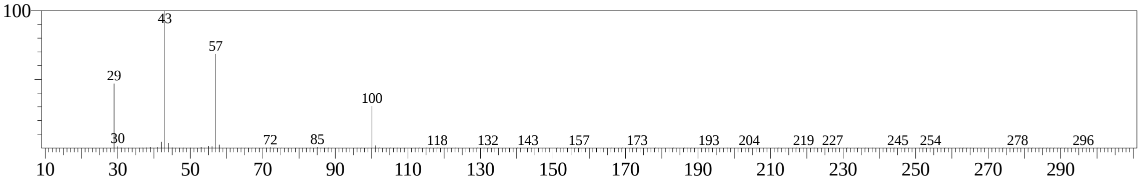

Hit#:1 Entry:1750 Library:NIST11s.lib
SI:94 Formula:C5H8O2 CAS:600-14-6 MolWeight:100 RetIndex:790
CompName:2,3-Pentanedione $$ Acetylpropionyl $$ 2,3-Pentadione $$ CH3C(O)C(O)C2H5 $$ Pentan-2,3-dione $$ Pentane-2,3-dione $$

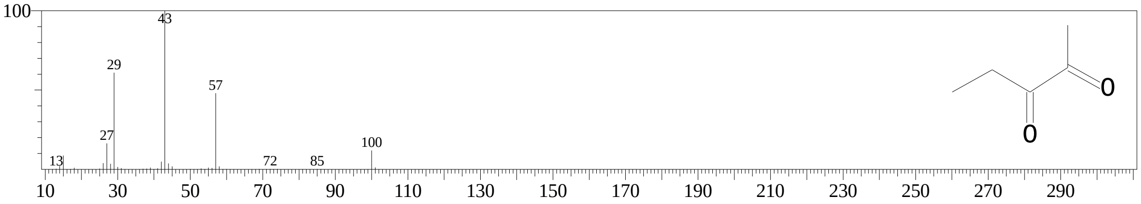

<< Target >>
Line#:2   R.Time:6.013(Scan#:905) MassPeaks:138
RawMode:Averaged 6.010-6.017(904-906) BasePeak:43.00(1778286)
BG Mode:Calc. from Peak Group 1 - Event 1

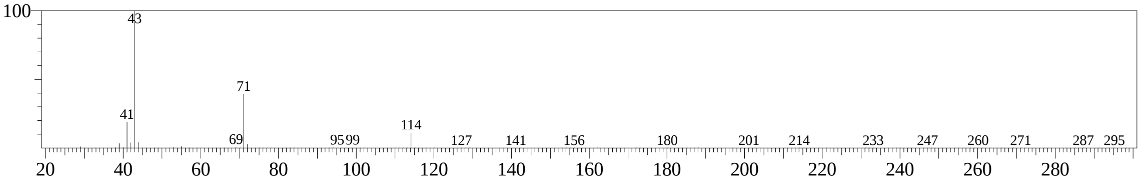

Hit#:1 Entry:3237 Library:NIST11s.lib
SI:96 Formula:C6H10O2 CAS:3848-24-6 MolWeight:114 RetIndex:890
CompName:2,3-Hexanedione $$ Acetylbutyryl $$ Methyl propyl diketone $$ 2,3-Hexandione $$

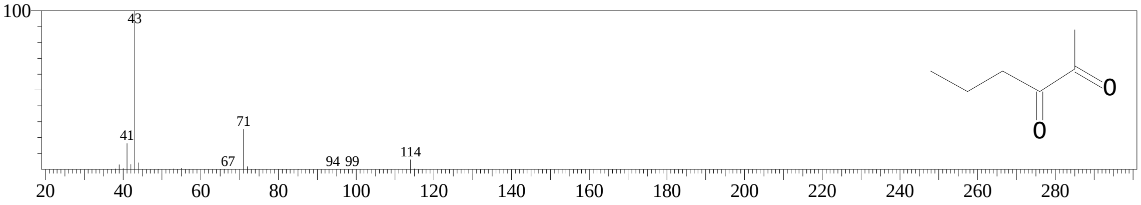

<< Target >>
Line#:3   R.Time:6.130(Scan#:940) MassPeaks:135
RawMode:Averaged 6.127-6.133(939-941) BasePeak:57.00(2245884)
BG Mode:Calc. from Peak Group 1 - Event 1

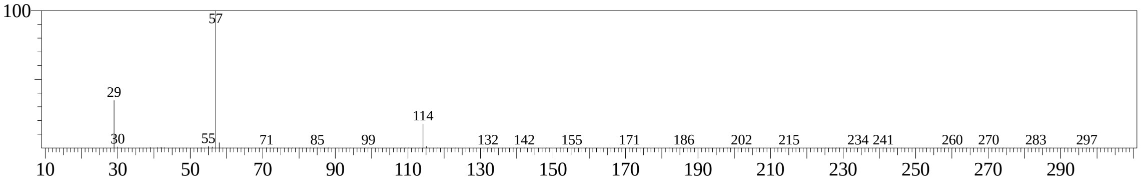

Hit#:1 Entry:3258 Library:NIST11s.lib
SI:95 Formula:C6H10O2 CAS:4437-51-8 MolWeight:114 RetIndex:890
CompName:3,4-Hexanedione $$ Bipropionyl $$ 3,4-Hexandione $$

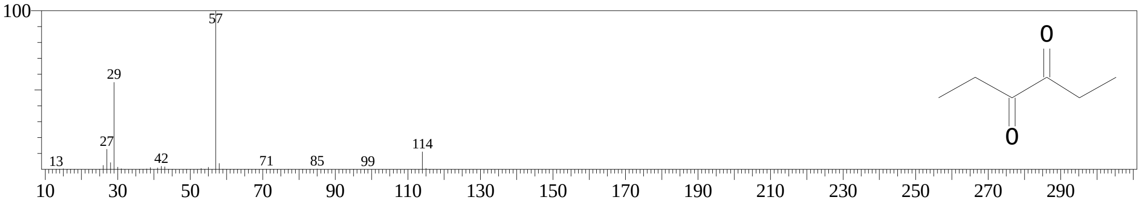

<< Target >>
Line#:4   R.Time:6.747(Scan#:1125) MassPeaks:121
RawMode:Averaged 6.743-6.750(1124-1126) BasePeak:57.05(1358820)
BG Mode:Calc. from Peak Group 1 - Event 1

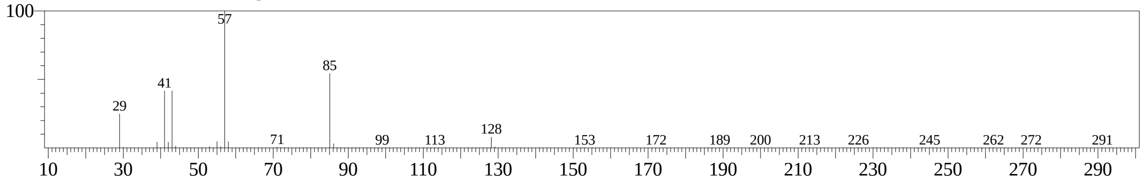

Hit#:1 Entry:7167 Library:NIST11.lib
SI:94 Formula:C7H12O2 CAS:96-04-8 MolWeight:128 RetIndex:989
CompName:Acetyl valeryl $$ 2,3-Heptanedione $$ Valerylacetyl $$

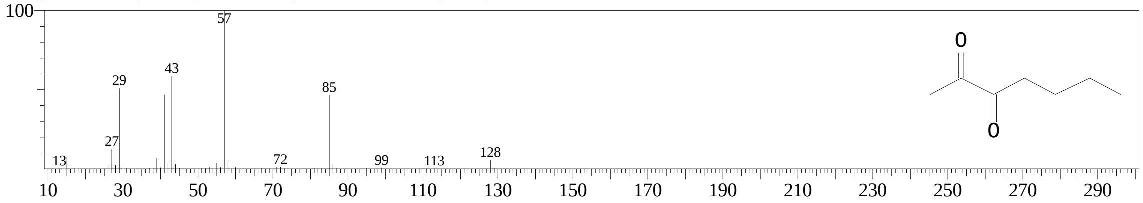

Supplement: Supplemental Information 1 [file peerj-03-1387-s006.zip › Raw data/diones standard mix GC-MS.pdf]
