# Supplementary material for: Ketoreductase TpdE from Rhodococcus jostii TMP1: characterization and application in the synthesis of chiral alcohols
Source: PeerJ. 2015 Nov 10;3:e1387. doi: 10.7717/peerj.1387 (PMC4647570; doi:10.7717/peerj.1387)

Sample Information

Analyzed by : Romualdas
Analyzed : 7/3/2013 5:36:51 PM
Sample Name : JoS_HpD
Injection Volume : 0.50
Data File : C:\GCMSsolution\Data\Project1\13.07.03_JoS_HpD.QGD
Method File : C:\GCMSsolution\Data\Project1\Standart_80_250_Col_1701.qgm
Tuning File : C:\GCMSsolution\System\Tune1\20130701.qgt
PASTABOS :
Modified : 7/3/2013 5:51:51 PM

Chromatogram JoS_HpD C:\GCMSsolution\Data\Project1\13.07.03_JoS_HpD.QGD

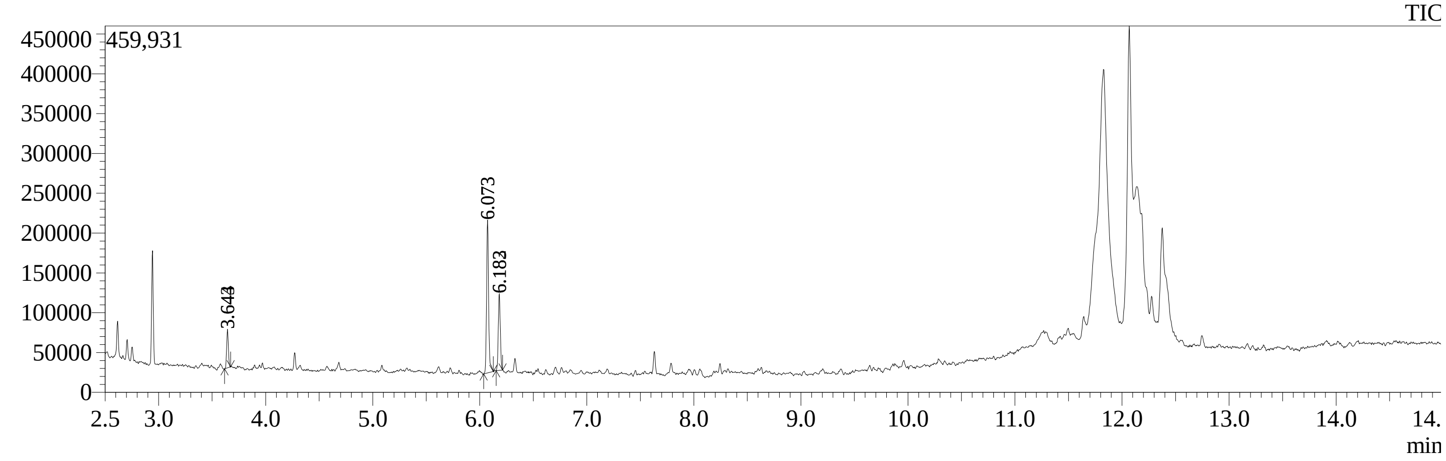

Peak Report TIC

Peak#	R.Time	Area%	Base m/z	Name
1	3.644	12.29	57.05	Acetyl valeryl (2,3-heptanedionas)
2	6.073	58.38	69.05	
3	6.182	29.32	69.05	
		100.00		

Spectrum

Line#:1 R.Time:3.643(Scan#:344)

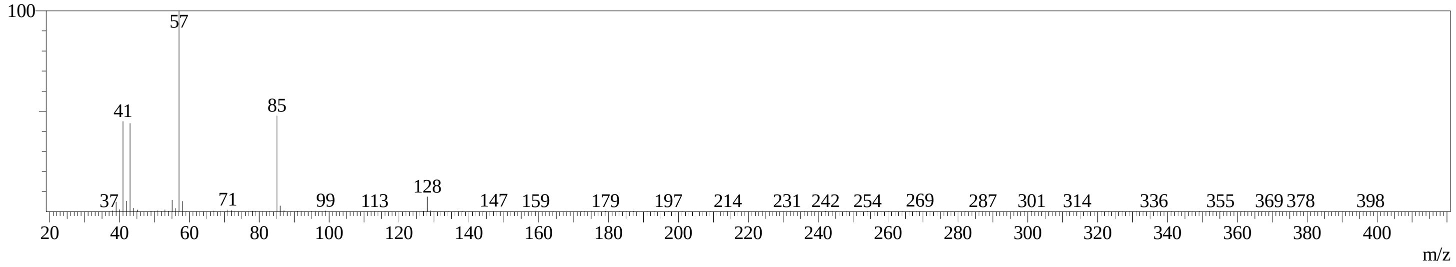

Line#:2 R.Time:6.073(Scan#:1073)

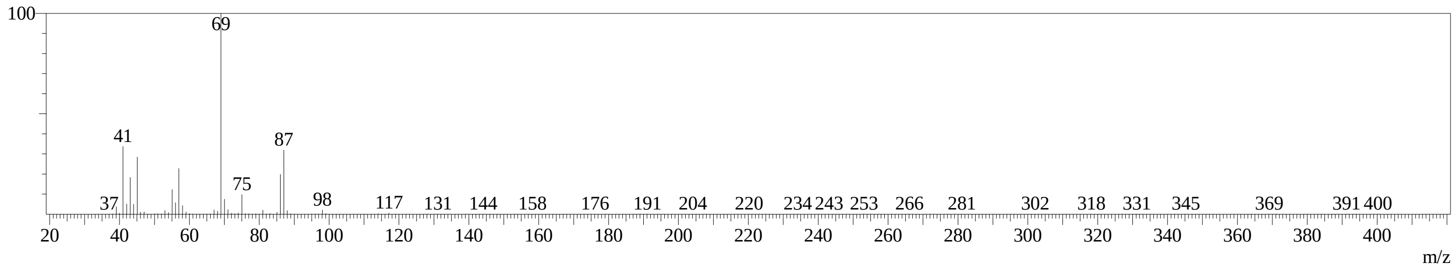

Line#:3 R.Time:6.183(Scan#:1106)

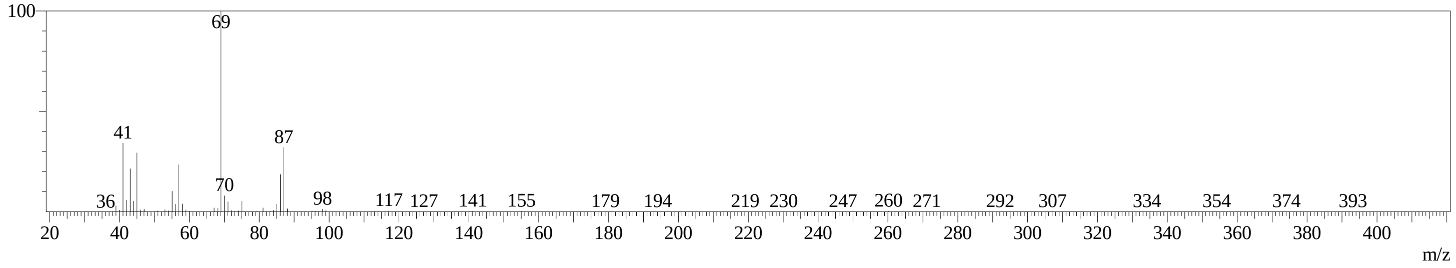

Supplement: Supplemental Information 1 [file peerj-03-1387-s006.zip › Raw data/Heptandione conv GC-MS.pdf]
