# Supplementary material for: Ketoreductase TpdE from Rhodococcus jostii TMP1: characterization and application in the synthesis of chiral alcohols
Source: PeerJ. 2015 Nov 10;3:e1387. doi: 10.7717/peerj.1387 (PMC4647570; doi:10.7717/peerj.1387)

Sample Information

Analyzed by : Romualdas
Analyzed : 7/3/2013 4:16:10 PM
Sample Name : JoS_23_HD
Injection Volume : 0.50
Data File : C:\GCMSsolution\Data\Project1\13.07.03_JoS_23_HD.QGD
Method File : C:\GCMSsolution\Data\Project1\Standart_80_250_Col_1701.qgm
Tuning File : C:\GCMSsolution\System\Tune1\20130701.qgt
PASTABOS :
Modified : 7/3/2013 4:31:10 PM

Chromatogram JoS_23_HD C:\GCMSsolution\Data\Project1\13.07.03_JoS_23_HD.QGD

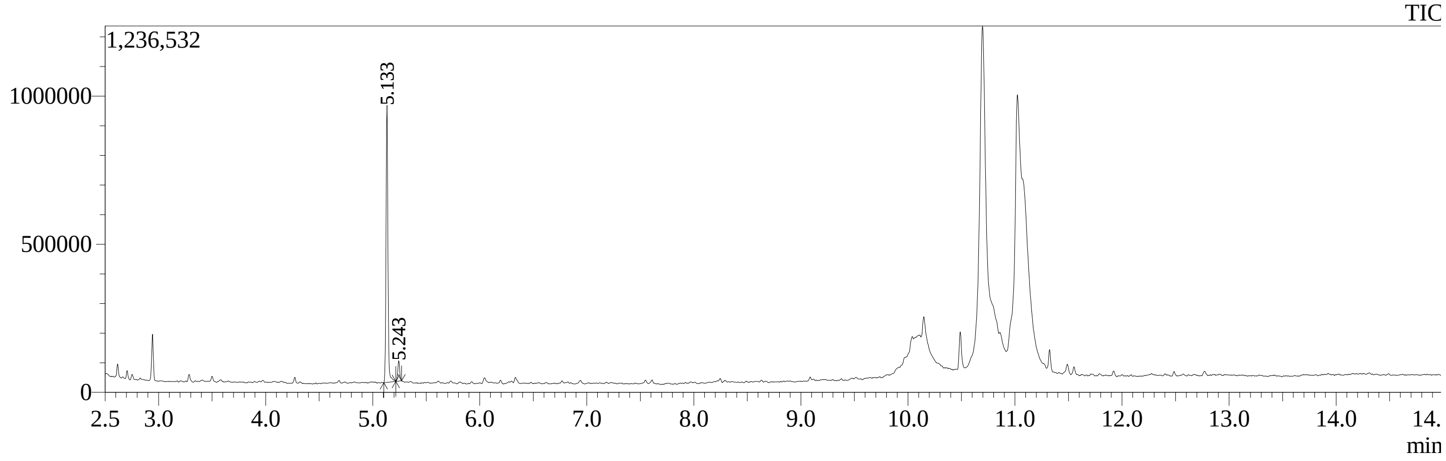

Peak Report TIC

Peak#	R.Time	Area%	Base m/z	Name
1	5.133	93.22	55.00	
2	5.243	6.78	55.00	
		100.00		

Spectrum

Line#:1 R.Time:5.133(Scan#:791)

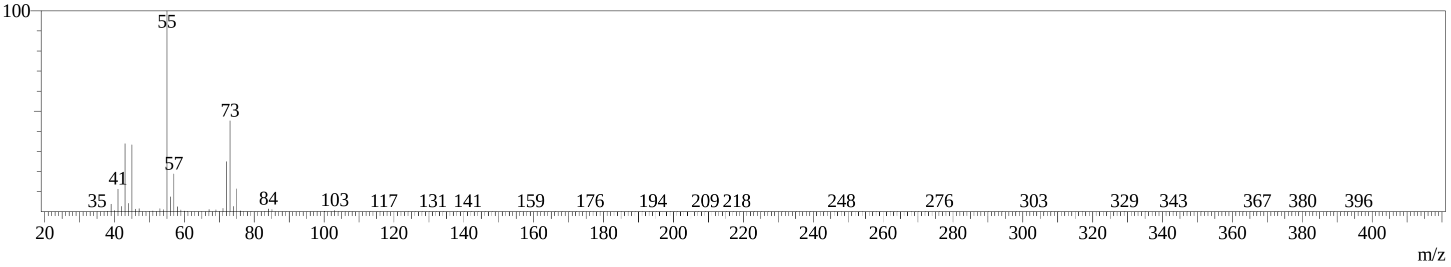

Line#:2 R.Time:5.243(Scan#:824)

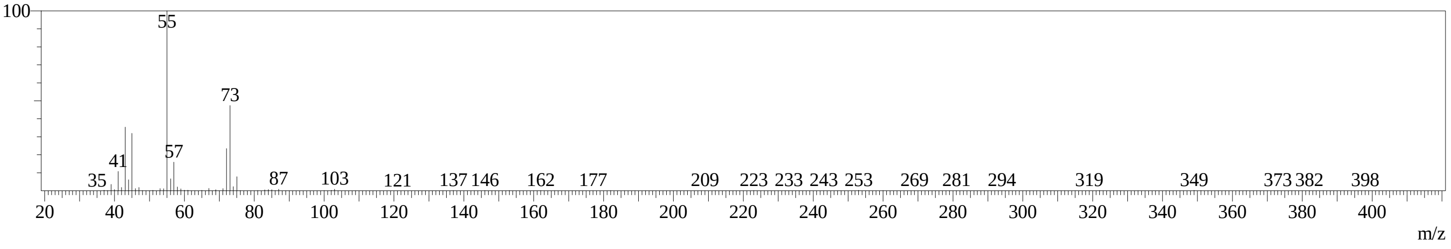

Supplement: Supplemental Information 1 [file peerj-03-1387-s006.zip › Raw data/Hexandione 2,3 conv GC-MS.pdf]
