# Supplementary material for: Ketoreductase TpdE from Rhodococcus jostii TMP1: characterization and application in the synthesis of chiral alcohols
Source: PeerJ. 2015 Nov 10;3:e1387. doi: 10.7717/peerj.1387 (PMC4647570; doi:10.7717/peerj.1387)

Sample Information

Analyzed by	: Romualdas
Analyzed	: 7/3/2013 4:36:12 PM
Sample Name	: JoS_34_HD
Injection Volume	: 0.50
Data File	: C:\GCMSsolution\Data\Project1\13.07.03_JoS_34_HD.QGD
Method File	: C:\GCMSsolution\Data\Project1\Standart_80_250_Col_1701.qgm
Tuning File	: C:\GCMSsolution\System\Tune1\20130701.qgt
PASTABOS :	
Modified	: 7/3/2013 4:51:12 PM

Chromatogram JoS_34_HD C:\GCMSsolution\Data\Project1\13.07.03_JoS_34_HD.QGD

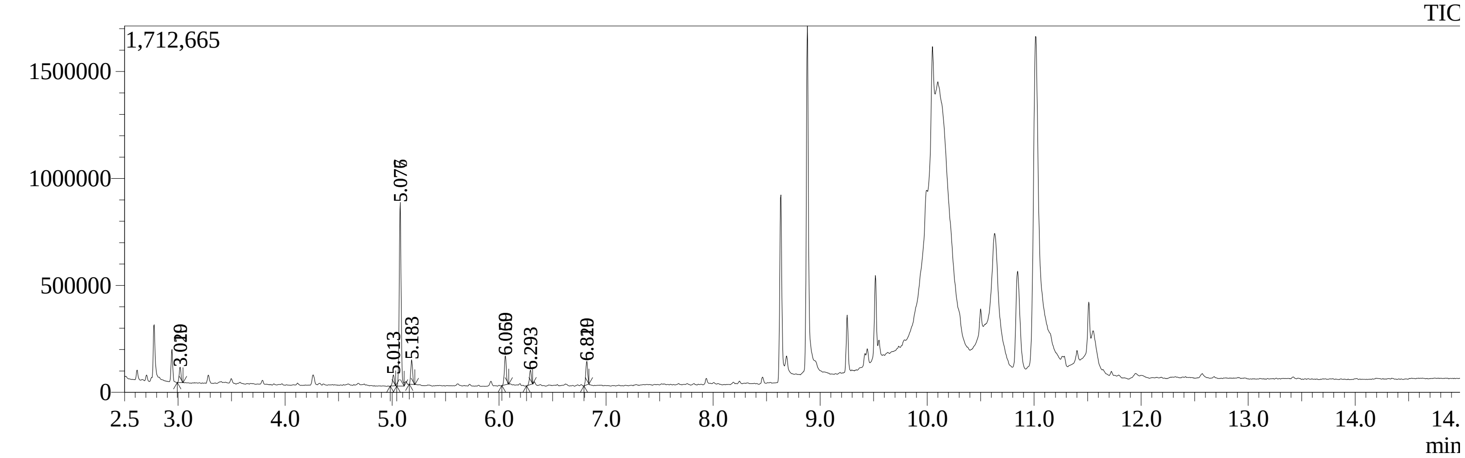

Peak Report TIC

Peak#	R.Time	Area%	Base m/z	Name
1	3.019	5.04	57.00	3,4-Hexanedione
2	5.013	3.54	102.05	
3	5.076	60.73	59.00	
4	5.183	7.91	59.00	
5	6.059	10.09	57.00	
6	6.293	4.80	57.00	
7	6.819	7.89	57.00	
		100.00		

Spectrum

Line#:1 R.Time:3.020(Scan#:157)

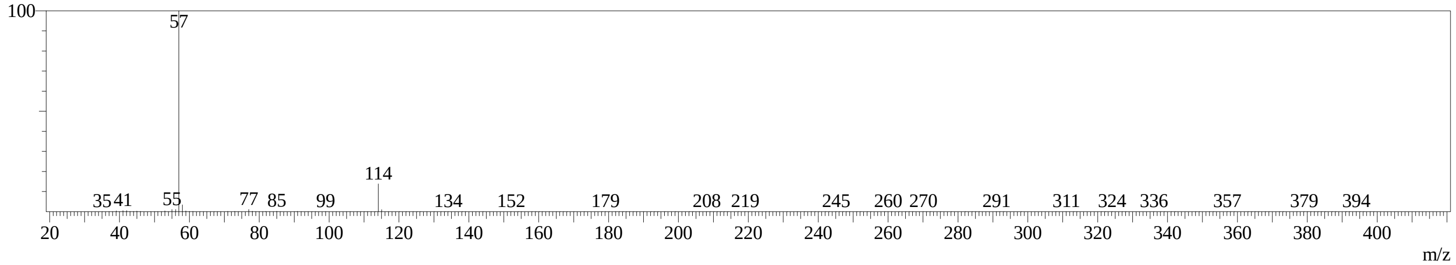

Line#:2 R.Time:5.013(Scan#:755)

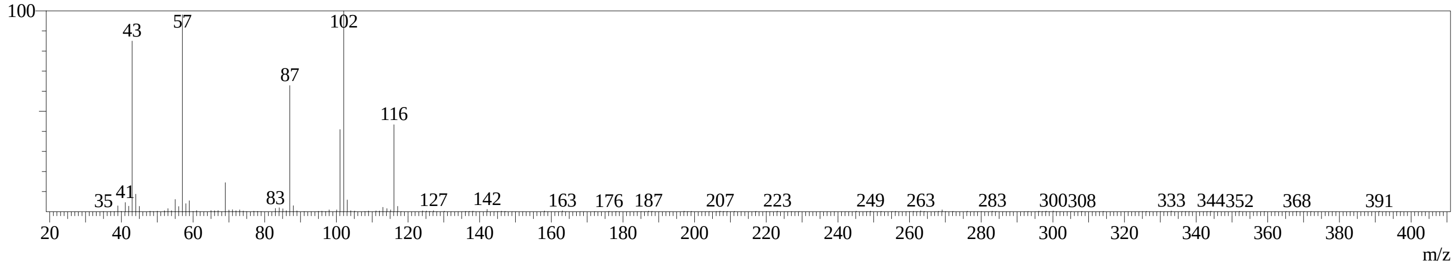

Line#:3 R.Time:5.077(Scan#:774)

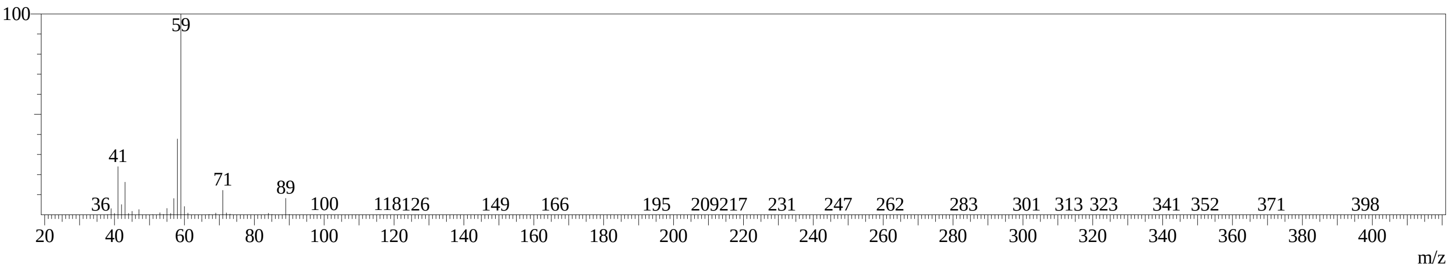

Line#:4 R.Time:5.183(Scan#:806)

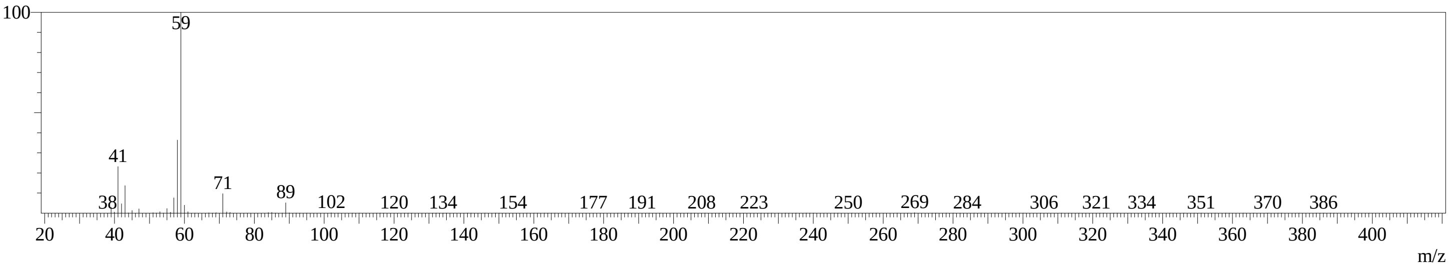

Line#:5 R.Time:6.060(Scan#:1069)

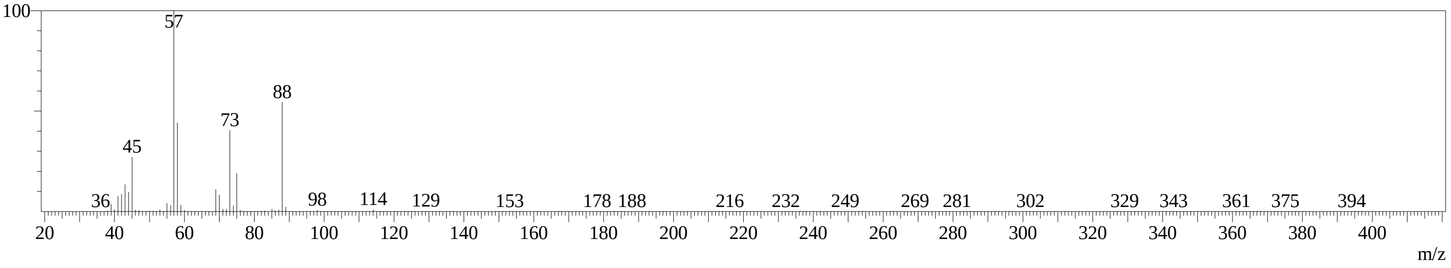

Line#:6 R.Time:6.293(Scan#:1139)

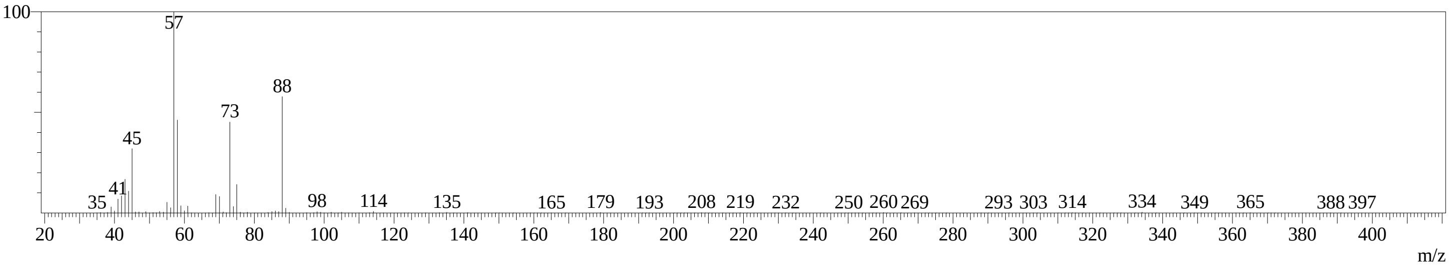

Line#:7 R.Time:6.820(Scan#:1297)

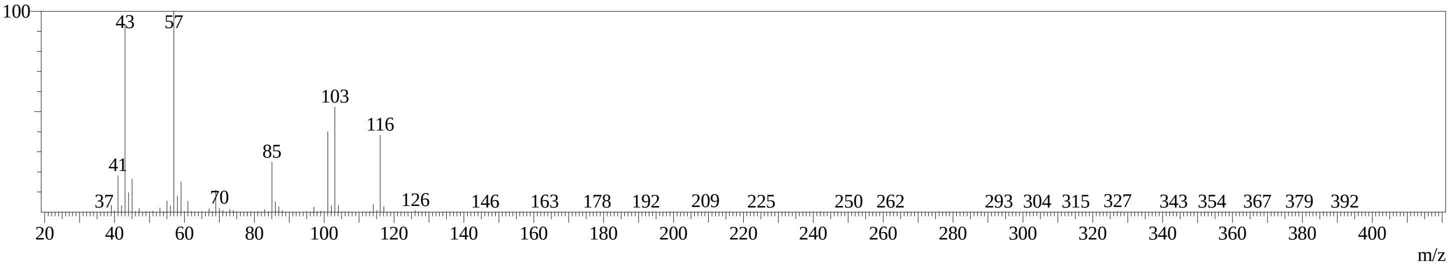

Supplement: Supplemental Information 1 [file peerj-03-1387-s006.zip › Raw data/Hexandione 3,4 conv GC-MS.pdf]
