# Supplementary material for: Ketoreductase TpdE from Rhodococcus jostii TMP1: characterization and application in the synthesis of chiral alcohols
Source: PeerJ. 2015 Nov 10;3:e1387. doi: 10.7717/peerj.1387 (PMC4647570; doi:10.7717/peerj.1387)

Sample Information

Analyzed by	: Romualdas
Analyzed	: 7/3/2013 5:16:35 PM
Sample Name	: JoS_DD
Injection Volume	: 0.50
Data File	: C:\GCMSsolution\Data\Project1\13.07.03_JoS_DD.QGD
Method File	: C:\GCMSsolution\Data\Project1\Standart_80_250_Col_1701.qgm
Tuning File	: C:\GCMSsolution\System\Tune1\20130701.qgt
PASTABOS :	
Modified	: 7/3/2013 5:31:35 PM

Chromatogram JoS_DD C:\GCMSsolution\Data\Project1\13.07.03_JoS_DD.QGD

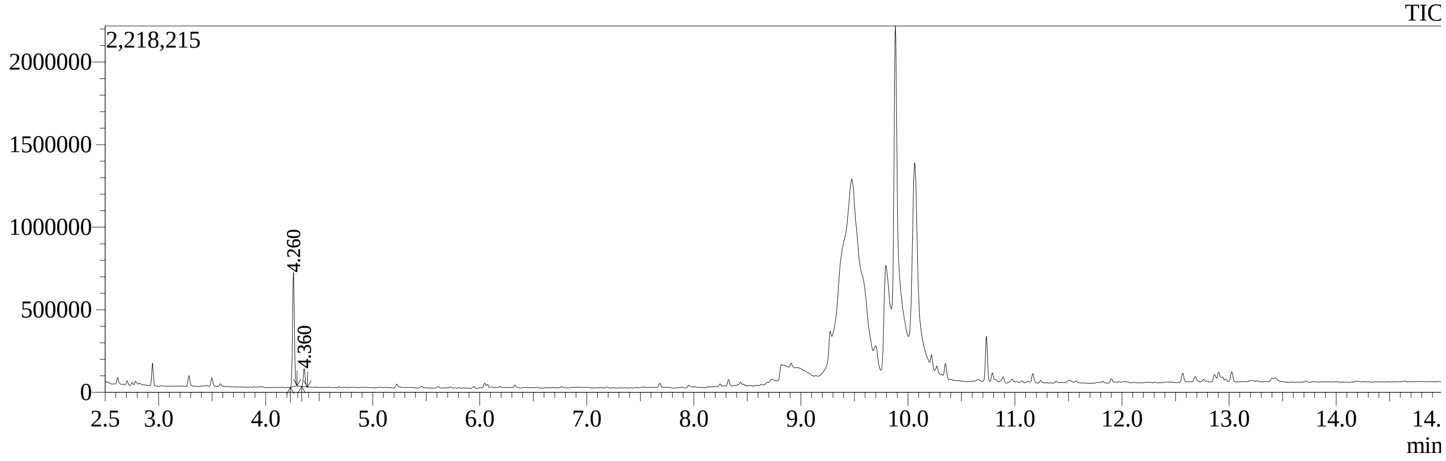

Peak Report TIC

Peak#	R.Time	Area%	Base m/z	Name
1	4.260	86.25	59.00	
2	4.360	13.75	59.05	
		100.00		

Spectrum

Line#:1 R.Time:4.260(Scan#:529)

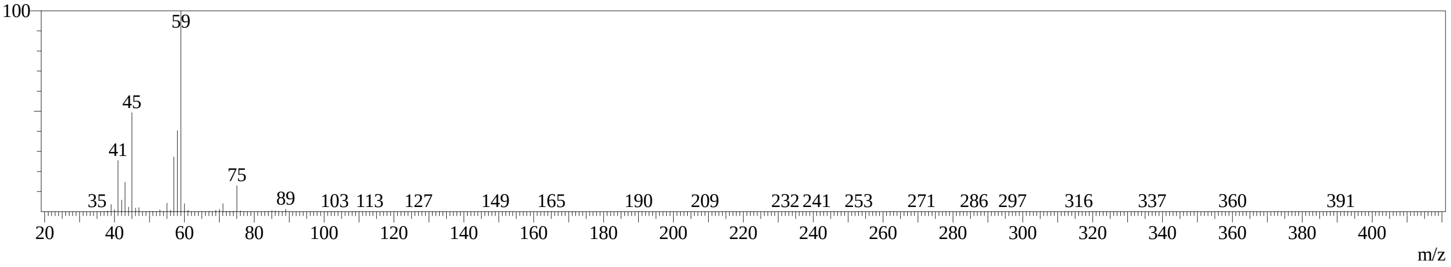

Line#:2 R.Time:4.360(Scan#:559)

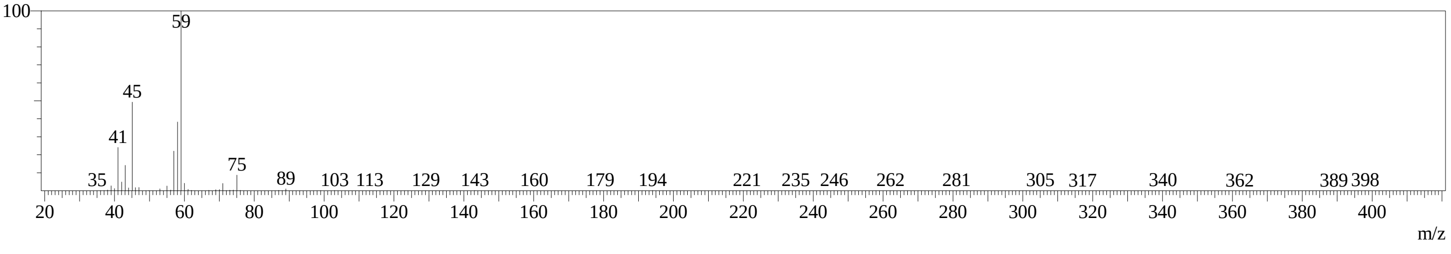

Supplement: Supplemental Information 1 [file peerj-03-1387-s006.zip › Raw data/Pentanedione conversion GC-MS.pdf]
